# Identifying Cases of Shoulder Injury Related to Vaccine Administration (SIRVA) in the United States: Development and Validation of a Natural Language Processing Method

**DOI:** 10.2196/30426

**Published:** 2022-05-24

**Authors:** Chengyi Zheng, Jonathan Duffy, In-Lu Amy Liu, Lina S Sy, Ronald A Navarro, Sunhea S Kim, Denison S Ryan, Wansu Chen, Lei Qian, Cheryl Mercado, Steven J Jacobsen

**Affiliations:** 1 Department of Research and Evaluation Kaiser Permanente Southern California Pasadena, CA United States; 2 Immunization Safety Office Centers for Disease Control and Prevention Atlanta, GA United States; 3 Kaiser Permanente South Bay Medical Center Harbor City, CA United States

**Keywords:** health, informatics, shoulder injury related to vaccine administration, SIRVA, natural language processing, NLP, causal relation, temporal relation, pharmacovigilance, electronic health records, EHR, vaccine safety, artificial intelligence, big data, population health, real-world data, vaccines

## Abstract

**Background:**

Shoulder injury related to vaccine administration (SIRVA) accounts for more than half of all claims received by the National Vaccine Injury Compensation Program. However, due to the difficulty of finding SIRVA cases in large health care databases, population-based studies are scarce.

**Objective:**

The goal of the research was to develop a natural language processing (NLP) method to identify SIRVA cases from clinical notes.

**Methods:**

We conducted the study among members of a large integrated health care organization who were vaccinated between April 1, 2016, and December 31, 2017, and had subsequent diagnosis codes indicative of shoulder injury. Based on a training data set with a chart review reference standard of 164 cases, we developed an NLP algorithm to extract shoulder disorder information, including prior vaccination, anatomic location, temporality and causality. The algorithm identified 3 groups of positive SIRVA cases (definite, probable, and possible) based on the strength of evidence. We compared NLP results to a chart review reference standard of 100 vaccinated cases. We then applied the final automated NLP algorithm to a broader cohort of vaccinated persons with a shoulder injury diagnosis code and performed manual chart confirmation on a random sample of NLP-identified definite cases and all NLP-identified probable and possible cases.

**Results:**

In the validation sample, the NLP algorithm had 100% accuracy for identifying 4 SIRVA cases and 96 cases without SIRVA. In the broader cohort of 53,585 vaccinations, the NLP algorithm identified 291 definite, 124 probable, and 52 possible SIRVA cases. The chart-confirmation rates for these groups were 95.5% (278/291), 67.7% (84/124), and 17.3% (9/52), respectively.

**Conclusions:**

The algorithm performed with high sensitivity and reasonable specificity in identifying positive SIRVA cases. The NLP algorithm can potentially be used in future population-based studies to identify this rare adverse event, avoiding labor-intensive chart review validation.

## Introduction

In 2017, shoulder injury related to vaccine administration (SIRVA) was officially added to the vaccine injury table by the National Vaccine Injury Compensation Program (VICP) [1-3]. The VICP defined SIRVA as shoulder pain and limited range of motion occurring after the administration of a vaccine intended for intramuscular administration in the upper arm. SIRVA is caused by an injury to the musculoskeletal structures of the shoulder (eg, tendons, ligaments, bursae). In 2019, the number of claims related to SIRVA rose to 55% of all claims received by VICP, which resulted in a payout of more than $200 million [4]. Meanwhile, there has been increasing debate on whether vaccination or vaccine can cause shoulder problems [5-7].

The debate is fueled by the lack of high-quality evidence from population-based studies [1,8-11]. Most SIRVA publications have been limited to case reports [12]. Based on reports filed in the Vaccine Adverse Event Reporting System (VAERS), one recent study examined cases of shoulder problems following influenza vaccine administration [13]. While VAERS data rely on spontaneous reporting and can be used for safety signal detection, comprehensive electronic medical record (EMR) data from integrated health care settings are better suited to calculate incidence rates, assess risk factors, or make causal inferences. One recent population-based study that used EMR data only examined one type of shoulder condition (subdeltoid bursitis) and one type of vaccination (influenza vaccine) [14].

Although EMR data provide unprecedented opportunities for research, much EMR data are stored as free text. Researchers frequently use manual chart review of medical records to acquire information that is not available from structured data in the EMR system. Because there are no defined diagnosis codes for SIRVA, SIRVA case identification and determination must be done by reviewing free-text clinical documents. Manual review is both costly and time consuming; this challenge is magnified with SIRVA. Because SIRVA occurs rarely, but shoulder problems are one of the most common musculoskeletal conditions, detecting SIRVA cases necessitates chart review of a significant number of medical records [11,14]. Compared with manual chart review of medical records, natural language processing (NLP) is more efficient and produces more consistent results [15,16]. For clinical research, NLP facilitates the identification and extraction of information unavailable or incomplete in structured data [17-19]. In vaccine safety studies, we have used NLP to identify 2 vaccine-related adverse events, anaphylaxis and local reaction [20,21]. Therefore, NLP has the potential to enable population-based SIRVA studies using EMR data.

Our objective was to develop an efficient SIRVA case-finding strategy using an NLP algorithm. We aimed to create and evaluate NLP components required for case identification, such as anatomic location, temporality, and causation. Furthermore, we sought to validate the SIRVA algorithm in a large, diverse vaccinated population.

## Methods

### Setting

This study was conducted at Kaiser Permanente Southern California (KPSC), an integrated health care system that provides prepaid comprehensive health care to more than 4.7 million racially, ethnically, and socioeconomically diverse members [22]. KPSC’s EMR system stores medical information about sociodemographics, utilization, diagnoses, laboratory tests, pharmacy use, membership history, and vaccination. This study was performed using structured data and free-text clinical notes from the EMR.

### Vaccinated Population With Presumptive Shoulder Injury

**Figure 1:**
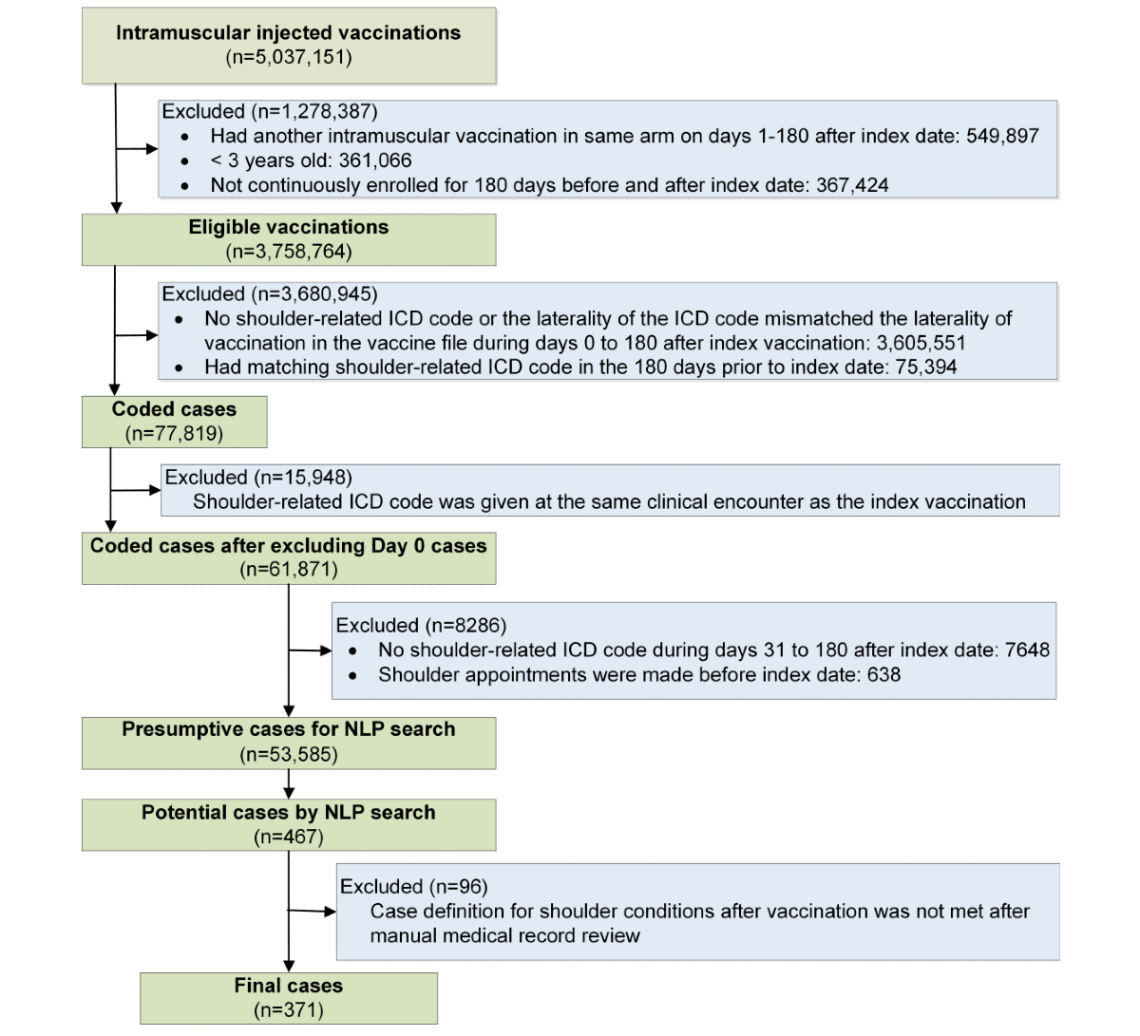
Flowchart showing selection of eligible vaccinations with presumptive shoulder injuries, application of natural language processing algorithm, and shoulder injury related to vaccine administration (SIRVA) case confirmation results (index date is vaccination date). ICD: International Classification of Diseases, 10th Revision, Clinical Modification; NLP: natural language processing.

The study was conducted among KPSC members aged 3 years or older who had at least 1 intramuscular vaccine administered in the arm between April 1, 2016, and December 31, 2017, within a KPSC facility (Figure 1). Each vaccination was specified by the members’ unique identifier, the vaccination date (index date: ie, day 0), and the laterality of vaccination. Membership was required for 180 days before and after the index date.

Among the vaccinated population described above, we identified members with a presumptive shoulder injury using *International Classification of Diseases, 10th Revision, Clinical Modification* (ICD-10-CM) codes (Multimedia Appendix 1) within 180 days after the index date; the laterality of the shoulder injury code had to match that of the vaccination. We excluded vaccinations if the members had a shoulder-related visit or had a shoulder injury code within 180 days before the index date.

On day 0, members could have had clinical visits with preexisting shoulder conditions and subsequently receive vaccinations. To exclude these day 0 preexisting conditions, we required at least 2 encounters on day 0, of which at least 1 of the latter encounters had to be an urgent care, emergency department, or virtual visit (email, telephone, or video encounter). We sorted day 0 encounters by their timestamps. Day 0 encounters were excluded if the first encounter on day 0 had a shoulder injury code or if the encounter occurred before vaccination. In order to exclude vaccine-related local reactions, one of the most common adverse events occurring shortly after vaccination, a shoulder injury code also needed to appear during days 31 to 180 postvaccination.

### SIRVA Case Definition

The VICP’s SIRVA case definition was created for medicolegal purposes [3]. To meet this case definition, a vaccine recipient must manifest all of the following: (1) pain and reduced range of motion are limited to the shoulder in which the intramuscular vaccine was administered, (2) pain occurs within 48 hours of vaccination, (3) no history of pain, inflammation, or dysfunction of the affected shoulder prior to vaccination that would explain the alleged condition, (4) no other condition or abnormality is present that would explain the patient’s symptoms, and (5) symptoms must last more than 6 months after vaccination [23].

Based on the VICP SIRVA case definition and other publications [1,8,13,14,24], we created a SIRVA case definition suitable for a population-based study using EMR data. A valid SIRVA case needed to meet 5 criteria: (1) damage to the shoulder region occurred and was confirmed by signs and symptoms (ie, pain, limited range of motion, weakness, and stiffness) and clinical diagnosis, (2) shoulder injury occurred in the same arm in which a vaccine was injected; (3) shoulder injury started within 7 days after vaccination, (4) vaccination was a possible cause of the shoulder injury and no other known causes were associated with the shoulder injury, and (5) shoulder injury lasted more than 30 days postvaccination.

### Subpopulation for Training and Validation of NLP Algorithm

**Figure 2:**
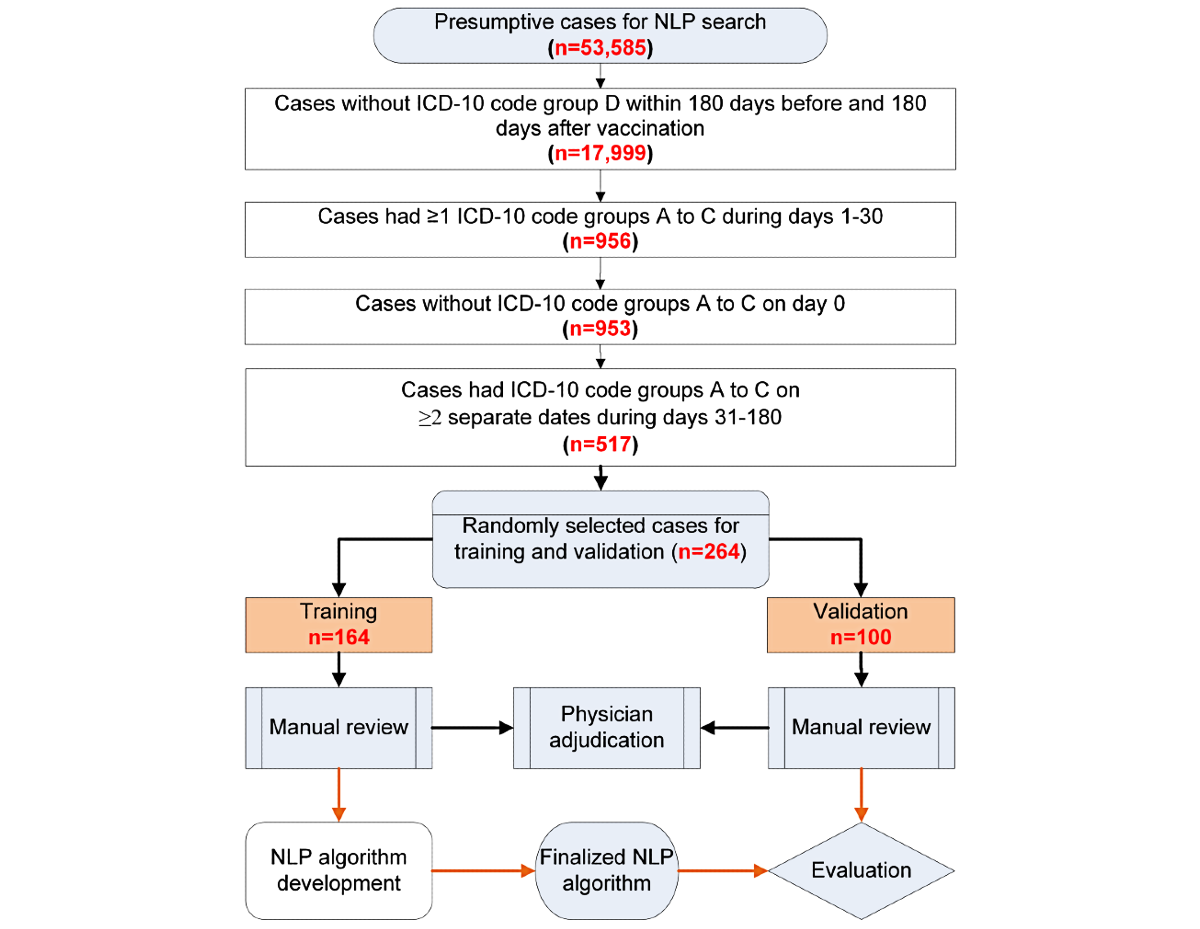
Flowchart to create data set for training and validation data sampling (group A: shoulder disorder diagnoses reported in shoulder injury related to vaccine administration [SIRVA] literature; group B: shoulder disorder diagnoses not previously reported in SIRVA literature; group C: shoulder symptom codes; group D: shoulder injury codes [ICD-10-CM chapter 19: Injury, poisoning and certain other consequences of external causes]). NLP: natural language processing; ICD-10: International Classification of Diseases, 10th Revision, Clinical Modification.

To increase the likelihood of including true SIRVA cases in the data sets used for training and validating the NLP algorithm, we applied additional criteria to the presumptive cases to define a subpopulation (n=517; Figure 2): (1) exclusion of cases with an external shoulder injury (eg, accident) code within 180 days before and 180 days after vaccination, (2) exclusion of cases with a shoulder injury code on day 0, (3) requirement of a shoulder injury code during days 1 to 30, and (4) requirement of a shoulder injury code on at least 2 different dates during days 31 to 180. The criteria were based on characteristics of chart-confirmed SIRVA cases from a prior study [14].

#### Training Data Set

From the substudy population described above, we selected a random sample for chart review. The NLP algorithm was built and refined based on incremental releases of training data [21]. In contrast to machine learning methods in which the model automatically updates its parameters based on training data, we manually created and updated the search queries based on training data. Once the NLP algorithm stabilized and achieved good performance, we stopped the training process. The final training dataset had 164 cases.

#### Validation Data Set

From the remaining cases in the substudy population (n=353), we randomly selected another 100 cases to form the validation dataset. The chart review results were used to evaluate the performance of the final NLP algorithm.

### Manual Chart Review

We created a chart review form based on the SIRVA case definition. Chart abstractors reviewed the medical records and recorded information on the abstraction form (Multimedia Appendix 2) using the REDCap (Research Electronic Data Capture) system [25]. The abstraction form was derived from a previous study of subdeltoid bursitis after vaccination but was expanded to include other shoulder disorder diagnoses [14]. The chart abstraction and adjudication processes were similar to those used in past vaccine safety studies [14,21]. An ascertainment period of 180 days after vaccination was used for both NLP and chart abstraction, allowing members sufficient time to seek medical care [14]. A second person reviewed each completed abstraction form for quality. A KPSC physician adjudicated the potential cases according to the SIRVA case definition for cases in which the chart reviewers had difficulty making a final assessment.

### NLP Terminology Development

NLP terminologies were derived from various data sources, including the clinical notes of the study participants, VAERS reports [26], ontologies (eg, Unified Medical Language System [27]), semantic lexicons (eg, WordNet [28]), and other online resources. We expanded the derived terminologies using various tools. We used Linguamatics I2E [29] to identify term variations including misspellings, morphological variants, and synonyms through I2E’s synonym discovery capability. We used word-embedding methods (fastText [30] and GloVe [31]) to find related terms not necessarily limited to synonyms. For instance, NLTK and fastText (from the Gensim package [32]) were used to train subword embedding models. Because our main interest was to identify rare terms to enrich our terminologies, we trained skip-gram models in fastText. The trained model was used to identify similar terms based on their contexts. For instance, the word “injury” has similar terms with various semantic meanings including accident, fall, laceration, overuse, trip, and sprain.

### NLP Indexing

The preprocessing steps included section detection, sentence separation, and tokenization (that is, segmenting text into linguistic units such as words and punctuation). For each token, the indexing process added annotations for matched concepts and general linguistic entities (eg, lexical chunks like noun or verb phrases). Additional annotations captured linguistic variations such as wildcard, substring, spelling correction, and morphological variation.

### NLP Search

**Figure 3:**
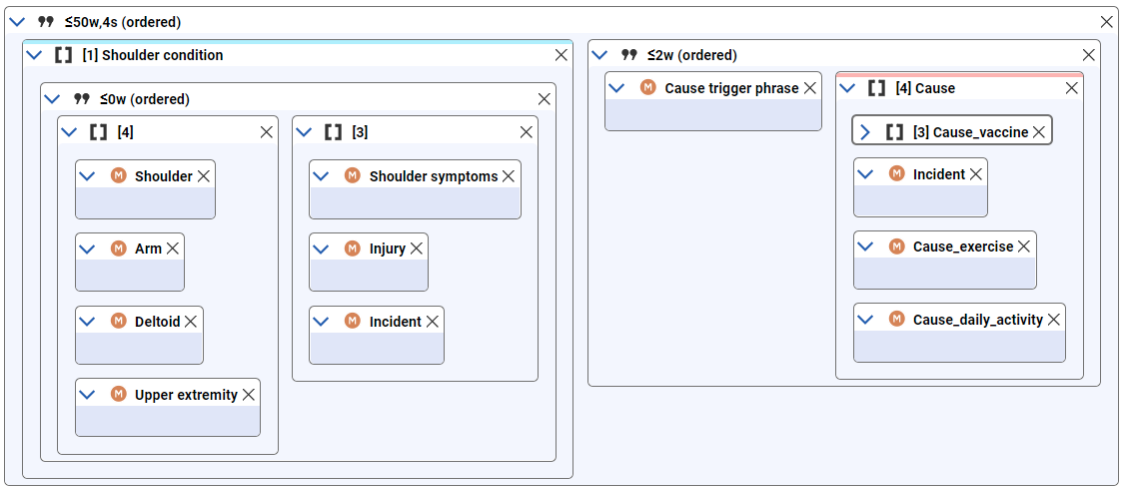
Cross-sentence search query example. This query searches over a span of 4 sentences (4s in diagram) with a maximum number of 50 words (≤50w in diagram) in between query items. There are 2 nested relationship queries inside the outermost relationship search. The first query searches for shoulder conditions, and the second query searches for causality statement. We removed other contextual query items from diagram due to space limitations. w: week; s: sentence.

We used a rule-based NLP algorithm for this study [15,21,33,34]. The NLP algorithm was developed to search each indexed note at different levels: section (eg, “past medical history”), intrasentence, and cross-sentence. A distance-based relationship detection algorithm was applied to relate terms to other terms based on the number of words or sentences between them, thereby associating shoulder injury with information on vaccination site, temporality, or causality (Figure 3). The relationship detection algorithm also allowed for terms to be specified as ordered or nested (eg, an inner relation is an element of an outer relation). We used negation algorithms similar to pyConText/NegEx [35] to identify negated, uncertain, and hypothetical statements. The relationship search identified 3 types of information associated with shoulder injury: anatomic, temporal, and causal.

The anatomic site relationship algorithm extracted the body location and laterality of the shoulder injury. For example, “left” and “arm” were identified as the laterality and body location of the shoulder injury, respectively, in the sentence “Patient has persistent pain in his left arm.”

The temporal relationship algorithm used linguistic terms, such as prepositions, to extract temporal relationships such as the onset date and duration associated with the vaccination event (eg, “for 2 months,” “over the past 2 weeks,” “since last Thursday”). Incomplete temporal information was inferred based on the note creation date. For example, dates with missing year information in clinical notes were assumed to occur near the note creation date. Additional details about the types of temporal expressions extracted by the NLP algorithm are available in Multimedia Appendix 3.

The causal relationship algorithm searched for possible causes of shoulder injury and classified them into 7 types (Table 1). The determination of causal relationships between cause and shoulder injury was made by lexical-syntactic rules based on more than 70 trigger terms (Multimedia Appendix 4). The terminologies for causes of shoulder injury other than vaccination are listed in Multimedia Appendix 5. Moreover, for each relationship search, we also extracted the vaccine name if available because multiple vaccines could be administered concomitantly or during follow-up.

**Table 1:** Types of causes associated with shoulder injuries.

Order	Type of cause	Description
1	Vaccination	Specific vaccine name or general vaccine terms
2	Accident	Accidents such as auto accident, fall, hit
3	Work	Work-related injury
4	Other medical conditions	Medical conditions that can cause shoulder injury such as arthritis or chest pain radiating to the shoulder
5	Exercise	Exercise or sports-related injury
6	Daily activity	Injuries occurred during other daily activities such as lifting groceries, overuse, or side sleeping
7	Unknown	Insidious or unknown cause

### NLP Case Classification

The final classification was based on the case definition described in the section “SIRVA case definition” by integrating vaccine, anatomic location, temporality, and causality information. Because our algorithm emphasized sensitivity, we captured additional probable and possible cases identified by NLP with weaker evidence as defined by the following 3 criteria. First, the vaccination cause was identified only by cross-sentence causal relationship search. For example, shoulder injury and vaccination were described in separate sentences: “Patient requesting an appointment for evaluation for left arm pain. States experiencing pain × 1 month s/p flu vaccine.” Second, vaccination was identified as a cause of shoulder injury 30 days or less after vaccination. Because causality was less likely to be documented when the visit date was further away from the onset date, vaccination may only be established as the cause of the shoulder injury within 30 days of vaccination, but not more than 30 days after vaccination. Third, the vaccine associated with shoulder injury documented in the clinical note did not match the vaccine recorded in the vaccination file. Positive cases that met the SIRVA case definition were further classified into 3 groups: definite if they met none of the 3 criteria; probable if they met only 1 of the 3 criteria; and possible if they met 2 or more of the 3 criteria.

### NLP Algorithm Performance

We evaluated the NLP algorithm’s accuracy in identifying SIRVA cases compared to the chart review reference standard in the validation dataset. We calculated sensitivity, specificity, positive predictive value, and negative predictive value and their 95% confidence intervals. Since the NLP algorithm could potentially be accurate in determining a case not to be SIRVA but based on an incorrect assessment of an individual component of the SIRVA case definition not being met, we also conducted an error analysis of cases in which there were discrepancies between the NLP algorithm and chart review for individual components of the case definition.

### Application of NLP Algorithm to Study Population and Chart Confirmation

The final NLP algorithm was applied to the broader study population of vaccinated persons with presumptive shoulder injury (based on codes) to identify potential SIRVA cases. We performed manual chart confirmation on all NLP-identified cases and calculated chart confirmation rates and their 95% confidence intervals.

We assembled the final group of SIRVA cases based on the chart review results. We calculated the time between vaccination and the first visit for a shoulder disorder in these SIRVA cases. We also examined the vaccination-related temporal and causal statements in the clinical notes of these SIRVA cases.

### Ethical Approval

The study was approved by the KPSC institutional review board (#4982), which waived the requirement for informed consent due to this being a data-only minimal risk study.

## Results

### Application of NLP Algorithm to Study Population

Out of 3,758,764 eligible vaccinations, we identified 77,819 records with a shoulder injury code (Figure 1). Among them, 16,048 had a code on day 0. After applying the day 0 inclusion criteria, the number of day 0 records remaining was 100. The NLP algorithm was applied to 53,585 cases with presumptive shoulder injury after vaccination.

### Validation Results

The NLP algorithm achieved perfect accuracy (100%) in identifying the 4 SIRVA cases from the validation dataset (n=100). However, the small number of positive cases resulted in wide confidence intervals for sensitivity and positive predictive value (39.6%-100.0%). Meanwhile, the confidence intervals for specificity and negative predictive value remained narrow (95.2%-100.0%).

Discrepancies between the NLP algorithm and chart review were investigated by component (Table 2). For laterality, discrepancies were typically due to conflicting evidence or documentation errors in the clinical notes themselves. For temporality, the NLP algorithm incorrectly assigned symptom onset when performing cross-sentence searches and incorrectly assigned injury duration based on incorrect laterality or capture of a resolved shoulder injury.

For causality, the NLP algorithm missed causes such as daily activity and accident and incorrectly identified the cause as unknown. These mistakes, however, had no bearing on the causality classification of whether or not they were vaccine-related. Furthermore, because a confirmed case must meet all of the elements of the case definition, inaccuracy in 1 element may not affect the overall accuracy of the SIRVA case classification.

**Table 2:** Error analyses on the validation dataset.

Clinical text examples and the causes of Natural Language Processing (NLP) errors		
**Error analysis on injury onset**		
	1	“She has chronic pain—neck, low back, B/L^a^ shoulders. She has fibromyalgia and also fell a few weeks ago which worsened her back pain.” NLP incorrectly associated the event (“fall”) that occurred “a few weeks ago” with the shoulder problem when performing a cross-sentence search.
	2	Prior condition reported on day 0 visit: “My left shoulder pain never went away despite still doing physical therapy and living on NSAIDs^b^. Now it is constant and much worse today.” NLP incorrectly captured “today” as the shoulder pain onset date when performing a cross-sentence search.
**Error analysis on injury duration**		
	3	On day 136, “States in past pain would travel to left shoulder causing numbness to left arm and lasting a few days but today denies any numbness.” NLP incorrectly identified the injury duration based on a resolved shoulder symptom.
**Error analysis on injury cause**		
	4	“...with 1 day of pain in the left arm and shoulder. Denies any injury. Did some lifting yesterday.” NLP identified the cause as unknown, failing to identify the possible cause (daily activity).
	5	“She has been working on the computer a lot. Overhead movement exacerbates the pain... No injury or trauma.” NLP identified the cause as unknown, failing to identify the possible cause (daily activity).
	6	“...who complains of left shoulder pain that started 3 weeks ago after vacuuming.” NLP identified the cause as unknown, failing to identify the possible cause (daily activity).
	7	“...likely subdeltoid bursitis and supraspinatus tendinopathy in the setting of DM^c^ likely from acute movement with pain when getting IV^d^ placed.” NLP identified the cause as unknown, failing to identify the possible cause (accident).
	8	“Patient reports left shoulder pain with movement; no trauma. Patient worked for years caring for young children and had to carry and lift them.” NLP identified the cause as unknown, failing to identify the possible cause (daily activity).

^a^B/L: bilateral.

^b^NSAIDs: Nonsteroidal anti-inflammatory drugs.

^c^DM: diabetes mellitus.

^d^IV: intravenous.

### NLP-Identified Potential SIRVA Cases

We applied the final NLP algorithm to the clinical notes of 53,585 presumptive shoulder injury cases. Among them, 99.9% (53,530/53,585) had at least 1 clinical note on days 0 to 180 after vaccination. The total number of clinical notes searched by NLP was 4,292,610. The average number of clinical notes per case was 80. The index size was around 50 gigabytes. The NLP algorithm identified shoulder injury in 46,086 records, and 96.5% of them had matched laterality compared to the vaccination files (Table 3). The NLP algorithm identified at least 1 cause for 55.0% (25,325/46,086) of the NLP-identified shoulder injury cases. The temporal relation search identified the onset date for 98.2% (45,252/46,086) of the NLP-identified shoulder injury cases. About 76.2% (35,135/46,086) of these NLP-identified shoulder injury cases had symptom duration of more than 30 days postvaccination. The number of potential SIRVA cases identified by the NLP algorithm was 467, classified into 291 definite, 124 probable, and 52 possible SIRVA cases.

**Table 3:** Number of cases identified by natural language processing (NLP) in the base study population (n=53,585).

Natural language processing–identified cases			n		%^a^ (n=53,585)		%^b^ (n=46,086)
Shoulder injury identified			46,086		86		—^c^
**Anatomic site**							
	Laterality identified	44,488		83		96.5	
	Laterality mismatch	1220		2.3		2.6	
**Causality**							
	Cause identified^d^	25,325		47.3		55.0	
	Cause identified^e^	19,039		35.5		41.3	
**Temporality**							
	Onset identified	45,252		84.4		98.2	
	Symptom duration >30 days postvaccination	35,135		65.6		76.2	
SIRVA^f^ cases			467		0.9		1

**^a^**Percentage of cases among the number of cases with shoulder injury diagnosis code (n=53,585).

^b^Percentage of cases among the number of natural language processing–identified shoulder injury cases (n=46,086).

^c^Not applicable.

^d^Includes unknown cause stated in the clinical notes.

^e^Excludes unknown cause stated in the clinical notes.

^f^SIRVA: shoulder injury related to vaccine administration.

### Final SIRVA Cases After Chart Review

We performed chart review on 467 NLP-identified SIRVA cases (Table 4). The chart confirmation rates were 95.5% (95% CI 92.5%-97.4%), 67.7% (95% CI 59.1%-75.3%), and 18.9% (95% CI 8.7%-30.8%) for the definite, probable, and possible groups, respectively. The final number of SIRVA cases was 371.

Among these 371 cases, the median times from vaccination to the first and last visit with a shoulder injury code were 43 days (IQR 21-79 days, range 0-180 days) and 127 days (IQR 77-162, range 31-180 days), respectively. The symptom onset occurred 2 or fewer days after vaccination in 93.5% (347/371) of cases and from 3 to 7 days after vaccination in 6.5% (24/371) of cases. Most cases (355/371, 95.7%) had explicit temporal statements on symptom onset in relation to vaccination. Examples included “L shoulder pain that started the day she got a flu shot” and “Right shoulder pain and neck stiffness since immunizations.” The symptom onset for the remaining cases (16/371, 4.3%) could be derived based on the date of clinical visit, symptom duration, and causality statement (eg, “Reports having R shoulder pain for last 2 months. Thought related to vaccine she received in R arm”). In 145 cases, there were explicit causal statements regarding the shoulder condition and the vaccination (eg, “status post vaccination—suspect rotator cuff irritation from vaccination itself”). Of those, 40 cases had mention of incorrect vaccine administration.

**Table 4:** Number of natural language processing–identified cases and chart-confirmed cases.

NLP^a^-identified group			NLP-identified	Chart confirmed		Confirmation rate (%)
Definite			291	278		95.5
**Probable**			124	84		67.7
	Cross-sentence causality	64		46	71.9	
	Vaccination cause identified ≤30 days after vaccination	41		26	63.4	
	Vaccine mismatch	19		12	63.2	
Possible			52	9		17.3
Total			467	371		79.4

^a^NLP: natural language processing.

## Discussion

### Principal Findings

SIRVA is a rare outcome after vaccination that does not have a specific diagnosis code, and it is impractical to conduct manual chart review to identify all SIRVA cases. We developed and validated an NLP algorithm to identify potential SIRVA cases with high accuracy. The only previous population-based study on SIRVA [14] was limited to shoulder bursitis after influenza vaccination. In that study, a random sample of 526 out of 1098 presumptive cases was chart reviewed to identify 12 subdeltoid bursitis cases attributed to vaccination. In this study, we included cases with all types of shoulder disorder diagnoses after vaccinations. Out of 53,585 presumptive cases, the NLP algorithm combined with manual chart review yielded 371 SIRVA cases. Among 3.8 million vaccinations, the rate of SIRVA in this study was around 1 per 10,000 vaccinations [12]. It should be noted that our SIRVA case definition was different from that of the VICP and other studies in terms of symptom onset, duration, and severity.

Although the NLP algorithm’s overall accuracy was high, some challenges remained with the laterality component, despite the addition of laterality information in ICD-10-CM coding. First, descriptions of symptom location may not be precise. For example, the arm could refer to the region from the shoulder joint to the elbow joint (upper arm) or further down to the wrist. Second, the laterality recorded in the vaccine file or documented in the clinical notes could be incorrect. These issues must be considered when conducting studies using anatomic and laterality information.

There were several lessons learned from the temporality component of the NLP algorithm. First, there could be documentation of multiple onset dates during the 180 days after vaccination. Second, the disease onset information was more likely to be incomplete or inaccurate when the onset date was in the distant past, which could make it difficult to determine the onset date if the clinical visit date was further away from the vaccination date. In this study, to maximize sensitivity, any potential case with an onset falling within the predefined onset window satisfied the onset criteria.

In our study, the causality component worked reasonably well in identifying vaccination-related causality statements. Although the provider or patient may have stated that the shoulder injury was vaccination-related, such statements do not provide definitive proof of causality. Because shoulder symptoms could have an insidious onset with multiple contributing factors, it was difficult to draw definitive conclusions about cause and effect. To improve specificity, we excluded cases with nonvaccination causes of shoulder injury. However, it was still challenging to identify nonvaccination causes. First, there were numerous causes of shoulder injuries. Second, some of the causes could also be the treatment for the shoulder problem. For example, exercise could be both the cause and the therapy plan for shoulder injuries. Third, the cause of shoulder injury was often not mentioned in the clinical notes. In this study, the NLP algorithm could not identify the cause in about half of the cases. Last, the cause of shoulder injury was often not described in the same sentence as the shoulder symptom. The cross-sentence relationship search increased the sensitivity but decreased specificity. Causal relations have been studied extensively in the NLP field [36], but only a few studies focused on health-related causal relations and were conducted using Twitter messages [37] and literature [38-40]. One study extracted causal relations from clinical text using 3 causal key phrases (because, due to, and secondary to) and discontinuation key phrases to detect adverse drug reactions in ambulatory notes and achieved high specificity (98%) but low sensitivity (31%) and positive predictive value (45%) [41].

SIRVA-related shoulder symptoms are common for other acute or chronic medical conditions with many possible causes. Correctly integrating the NLP-identified laterality, temporality, and causality information is nontrivial. For the same patient, different clinical encounters could attribute the shoulder injury to different causes. In this study, we made patient-level classifications by using the information identified from all the components from all the notes. The combination of information across multiple notes increased the sensitivity of finding SIRVA cases but reduced the specificity since the NLP algorithm could misinterpret unrelated information extracted from multiple notes.

Because we tailored the NLP algorithm to emphasize sensitivity, the confirmation rates were low in the probable (67.7%) and possible (17.3%) groups. However, since SIRVA is a rare event, manual review of all the probable and possible cases was feasible in this study. In future studies, instead of categorizing the NLP output based on the strength of evidence, a machine learning model could be built on top of the NLP outputs [15] to further improve accuracy and develop thresholds. The SIRVA cases identified in this study could also serve as training data for a machine learning algorithm.

### Limitations

This study had some potential limitations. We were unable to apply the algorithm to all the eligible vaccinations (n=3,758,764) due to time and resource restrictions. Our study population was limited to vaccinated cases with a diagnosis code for shoulder injury. However, loss of sensitivity is expected to be minimal since we used a comprehensive list of codes. Additionally, shoulder injuries can last a long time and are often accompanied by repeated visits. The 6-month lookback window used in this study may not have been sufficient to remove preexisting shoulder conditions. Failure to exclude prior shoulder conditions could reduce the specificity of the NLP algorithm. In our vaccine-related local reaction study [20], most people diagnosed with a presumptive code of interest on day 0 had symptom onset before vaccination. In this study, we excluded most cases with a shoulder injury code on day 0. Further research is needed to study the association between SIRVA and day 0 shoulder injury codes. Finally, because our method was tailored to this specific outcome after vaccination, its generalizability for use with other outcomes is unclear.

### Conclusions

We developed and validated an NLP algorithm to identify potential SIRVA cases among vaccinated persons with presumptive shoulder injury. The algorithm achieved high sensitivity and reasonable specificity. The NLP algorithm can potentially be used in future population-based studies to identify this rare adverse event, avoiding labor-intensive chart review validation.

## Appendix

Multimedia Appendix 1 International Classification of Diseases, 10th Revision, Clinical Modification code groups for identifying presumptive shoulder injury cases.

Multimedia Appendix 2 Chart review abstraction form.

Multimedia Appendix 3 Sample extracted temporal expressions.

Multimedia Appendix 4 Trigger phrases for identifying causal relationships.

Multimedia Appendix 5 Terminology for causes of shoulder injury other than vaccination.

## Abbreviations

CDC: Centers for Disease Control and Prevention
EMR: electronic medical record
KPSC: Kaiser Permanente Southern California
ICD-10-CM: International Classification of Diseases, 10th Revision, Clinical Modification
NLP: natural language processing
REDCap: Research Electronic Data Capture
SIRVA: shoulder injury related to vaccine administration
VAERS: Vaccine Adverse Event Reporting System
VICP: National Vaccine Injury Compensation Program
