# Supplementary material for: Identifying Cases of Shoulder Injury Related to Vaccine Administration (SIRVA) in the United States: Development and Validation of a Natural Language Processing Method
Source: JMIR Public Health Surveill. 2022 May 24;8(5):e30426. doi: 10.2196/30426 (PMC9175103; doi:10.2196/30426)
Supplement: Multimedia Appendix 1 [file publichealth_v8i5e30426_app1.docx]

**Appendix 1. ICD-10-CM Code Groups for Identifying Presumptive Shoulder Injury Cases**

**Identifying Cases of Shoulder Injury Related to Vaccine Administration (SIRVA) in the United States: Development and Validation of a Natural Language Processing Method**

Chengyi Zheng^1^, PhD, Jonathan Duffy^2^, MD, In-Lu Amy Liu^1^, MS, Lina S. Sy^1^, MPH, Ronald A. Navarro^3^, MD, Sunhea S. Kim^1^, MPH, Denison S. Ryan^1^, MPH, Wansu Chen^1^, PhD, Lei Qian^1^, PhD, Cheryl Mercado^1^, MPH, Steven J. Jacobsen^1^, MD, PhD

^1^ Department of Research & Evaluation, Kaiser Permanente Southern California, Pasadena, California, USA

^2^ Immunization Safety Office, Centers for Disease Control and Prevention, Atlanta, GA, USA

^3^ Kaiser Permanente South Bay Medical Center, Harbor City, California

**Corresponding Author:**

Chengyi Zheng, PhD

Department of Research and Evaluation, Kaiser Permanente Southern California

100 S Los Robles Ave, 2nd Floor,

Pasadena, CA 91101

United States

Phone: 1 626 986 8665

Email: Chengyi.X.Zheng@kp.org

**ICD-10-CM Code Groups for Identifying Presumptive Shoulder Injury Cases**

| **Code group** | **Description** |
| --- | --- |
| ICD-10 code group A | shoulder disorder diagnoses reported in the SIRVA literature |
| ICD-10 code group B | other shoulder disorder diagnoses not previously reported in the SIRVA literature |
| ICD-10 code group C | shoulder symptom codes |
| ICD-10 code group D | shoulder injury codes (ICD-10-CM chapter 19: Injury, poisoning and certain other consequences of external causes) |

ICD-10-CM = International Classification of Diseases, 10^th^ Revision, Clinical Modification.

The complete list of ICD-10 codes for each code group is listed below.

**Table S1.1. Shoulder Disorder ICD-10-CM Codes Reported in the SIRVA Literature (ICD-10 Code Group A)**

|  |  | | **Laterality** | | |
| --- | --- | --- | --- | --- | --- |
| **Code** | | **Diagnosis** | **Left** | **Right** | **Unspecified** |
| **Shoulder bursitis diagnosis codes** | | |  |  |  |
| M75.5X | | Bursitis of Shoulder | M75.52 | M75.51 | M75.50 |
| M71.31 | | Other bursal cyst, shoulder | M71.312 | M71.311 | M71.319 |
| M71.81 | | Other specified bursopathies, shoulder | M71.812 | M71.811 | M71.819 |
| **Shoulder diagnosis codes (not bursitis)** | | |  |  |  |
| M02.21X | | Postimmunization arthropathy | M02.212 | M02.211 | M02.219 |
| M13.11X | | Monoarthritis, not elsewhere classified, shoulder | M13.112 | M13.111 | M13.119 |
| M13.81X | | Other specified arthritis, shoulder | M13.812 | M13.811 | M13.819 |
| M25.41X | | Effusion of shoulder | M25.412 | M25.411 | M25.419 |
| M65.11X | | Other infective tenosynovitis, shoulder | M65.112 | M65.112 | M65.119 |
| M65.81X | | Other synovitis and tenosynovitis, shoulder | M65.812 | M65.811 | M65.819 |
| M67.31 | | Transient synovitis, shoulder | M67.312 | M67.311 | M67.319 |
| M67.81 | | Other specified disorders of synovium and tendon, shoulder | M67.812,  M67.814 | M67.811,  M67.813 | M67.819 |
| M67.91 | | Unspecified disorder of synovium and tendon, shoulder | M67.912 | M67.911 | M67.919 |
| M67.92 | | Unspecified disorder of synovium and tendon, upper arm | M67.922 | M67.921 | M67.929 |
| M75.0X | | Adhesive capsulitis/frozen shoulder | M75.02 | M75.01 | M75.00 |
| M75.1XX | | Tears of rotator cuff | M75.102,  M75.112,  M75.122 | M75.101,  M75.111,  M75.121 | M75.100,  M75.110,  M75.120 |
| M75.2X | | Bicipital tendinitis | M75.22 | M75.21 | M75.20 |
| M75.3X | | Calcific tendinitis of shoulder | M75.32 | M75.31 | M75.30 |
| M87.01X | | Idiopathic asceptic necrosis of shoulder | M87.012 | M87.011 | M87.019 |
| M87.02X | | Idiopathic asceptic necrosis of humerus | M87.022 | M87.021 | M87.029 |
| M87.11X | | Osteonecrosis due to drugs, shoulder | M87.112 | M87.111 | M87.119 |
| M87.12X | | Osteonecrosis due to drugs, humerus | M87.122 | M87.121 | M87.129 |
| M87.21X | | Osteonecrosis due to previous trauma, shoulder | M87.212 | M87.211 | M87.219 |
| M87.22X | | Osteonecrosis due to previous trauma, humerus | M87.222 | M87.221 | M87.229 |
| M87.31X | | Other secondary osteonecrosis, shoulder | M87.312 | M87.311 | M87.319 |
| M87.32X | | Other secondary osteonecrosis, humerus | M87.322 | M87.321 | M87.329 |
| M87.81X | | Other osteonecrosis, shoulder | M87.812 | M87.811 | M87.819 |
| M87.82X | | Other osteonecrosis, humerus | M87.822 | M87.821 | M87.829 |
| M89.31X | | Hypertrophy of bone, shoulder | M89.312 | M89.311 | M89.319 |
| M89.51X | | Osteolysis, shoulder | M89.512 | M89.511 | M89.519 |
| M89.52X | | Osteolysis, upper arm | M89.522 | M89.521 | M89.529 |
| S46.00X | | Unspecified injury of muscle(s) and tendon(s) of rotator cuff | S46.002* | S46.001* | S46.009* |
| S46.09X | | Other injury of muscle(s) and tendon(s) of rotator cuff | S46.092* | S46.091* | S46.099* |
| S40.01X | | Contusion of shoulder | S40.012* | S40.011* | S40.019* |
| S46.01X | | Strain of muscle(s) and tendon(s) of the rotator cuff | S46.012* | S46.011* | S46.019* |

**Table S1.2. Shoulder Disorder ICD-10-CM Codes not Previously Reported in the SIRVA Literature (ICD-10 Code Group B)**

|  |  | **Laterality** | | |
| --- | --- | --- | --- | --- |
| **Code** | **Diagnosis** | **Left** | **Right** | **Unspecified** |
| M14.61 | Charcots joint, shoulder | M14.612 | M14.611 | M14.619 |
| M21.21 | Flexion deformity, shoulder | M21.212 | M21.211 | M21.219 |
| M24.11 | Other articular cartilage disorders, shoulder | M24.112 | M24.111 | M24.119 |
| M24.21 | Disorder of ligament, shoulder | M24.212 | M24.211 | M24.219 |
| M24.41 | Recurrent dislocation, shoulder | M24.412 | M24.411 | M24.419 |
| M24.51 | Contracture, shoulder | M24.512 | M24.511 | M24.519 |
| M24.61 | Ankylosis, shoulder | M24.612 | M24.611 | M24.619 |
| M24.81 | Other specific joint derangements of shoulder, not elsewhere classified | M24.812 | M24.811 | M24.819 |
| M25.01 | Hemarthrosis, shoulder | M25.012 | M25.011 | M25.019 |
| M25.11 | Fistula, shoulder | M25.112 | M25.111 | M25.119 |
| M25.21 | Flail joint, shoulder | M25.212 | M25.211 | M25.219 |
| M25.31 | Other instability, shoulder | M25.312 | M25.311 | M25.319 |
| M25.71 | Osteophyte, shoulder | M25.712 | M25.711 | M25.719 |
| M25.81 | Other specified joint disorders, shoulder | M25.812 | M25.811 | M25.819 |
| M60.21 | Foreign body granuloma of soft tissue, not elsewhere classified, shoulder | M60.212 | M60.211 | M60.219 |
| M60.22 | Foreign body granuloma of soft tissue, not elsewhere classified, upper arm | M60.222 | M60.221 | M60.229 |
| M60.81 | Other myositis shoulder | M60.812 | M60.811 | M60.819 |
| M60.82 | Other myositis upper arm | M60.822 | M60.821 | M60.829 |
| M61.01 | Myositis ossificans traumatica, shoulder | M61.012 | M61.011 | M61.019 |
| M61.02 | Myositis ossificans traumatica, upper arm | M61.022 | M61.021 | M61.029 |
| M61.41 | Other calcification of muscle, shoulder | M61.412 | M61.411 | M61.419 |
| M61.42 | Other calcification of muscle, upper arm | M61.422 | M61.421 | M61.429 |
| M61.51 | Other ossification of muscle, shoulder | M61.512 | M61.511 | M61.519 |
| M61.52 | Other ossification of muscle, upper arm | M61.522 | M61.521 | M61.529 |
| M62.01 | Separation of muscle (nontraumatic), shoulder | M62.012 | M62.011 | M62.019 |
| M62.02 | Separation of muscle (nontraumatic), upper arm | M62.022 | M62.021 | M62.029 |
| M62.11 | Other rupture of muscle (nontraumatic), shoulder | M62.112 | M62.111 | M62.119 |
| M62.12 | Other rupture of muscle (nontraumatic), upper arm | M62.122 | M62.121 | M62.129 |
| M62.21 | Nontraumatic ischemic infarction of muscle, shoulder | M62.212 | M62.211 | M62.219 |
| M62.22 | Nontraumatic ischemic infarction of muscle, upper arm | M62.222 | M62.221 | M62.229 |
| M62.41 | Contracture of muscle, shoulder | M62.412 | M62.411 | M62.419 |
| M62.41 | Contracture of muscle, upper arm | M62.422 | M62.421 | M62.429 |
| M62.51 | Muscle wasting and atrophy, not elsewhere classified, shoulder | M62.512 | M62.511 | M62.519 |
| M62.52 | Muscle wasting and atrophy, not elsewhere classified, upper arm | M62.522 | M62.521 | M62.529 |
| M66.11 | Rupture of synovium, shoulder | M66.112 | M66.111 | M66.119 |
| M66.21 | Spontaneous rupture of extensor tendons, shoulder | M66.212 | M66.211 | M66.219 |
| M66.22 | Spontaneous rupture of extensor tendons, upper arm | M66.222 | M66.221 | M66.229 |
| M66.31 | Spontaneous rupture of flexor tendons, shoulder | M66.312 | M66.311 | M66.319 |
| M66.32 | Spontaneous rupture of flexor tendons, upper arm | M66.322 | M66.321 | M66.329 |
| M66.81 | Spontaneous rupture of other tendons, shoulder | M66.812 | M66.811 | M66.819 |
| M66.82 | Spontaneous rupture of other tendons, upper arm | M66.822 | M66.821 | M66.829 |
| M67.21 | Synovial hypertrophy, not elsewhere classified, shoulder | M67.212 | M67.211 | M67.219 |
| M67.22 | Synovial hypertrophy, not elsewhere classified, upper arm | M67.222 | M67.221 | M67.229 |
| M67.41 | Ganglion, shoulder | M67.412 | M67.411 | M67.419 |
| M70.81 | Other soft tissue disorders related to use, overuse and pressure of shoulder | M70.812 | M70.811 | M70.819 |
| M70.91 | Unspecified soft tissue disorder related to use, overuse and pressure of shoulder | M70.912 | M70.911 | M70.919 |
| M75.4 | Impingement syndrome of shoulder | M75.42 | M75.41 | M75.40 |
| M75.8 | Other shoulder lesions | M75.82 | M75.81 | M75.80 |
| M75.9 | Shoulder lesion, unspecified | M75.92 | M75.91 | M75.90 |
| M79.A1 | Nontraumatic compartment syndrome of upper extremity | M79.A12 | M79.A11 | M79.A19 |
| **Other osteopathies** | | | | |
| M89.01 | Algoneurodystrophy, shoulder | M89.012 | M89.011 | M89.019 |
| M89.02 | Algoneurodystrophy, upper arm | M89.022 | M89.021 | M89.029 |
| M89.71 | Major osseous defect, shoulder region | M89.712 | M89.711 | M89.719 |
| M89.72 | Major osseous defect, humerus | M89.722 | M89.721 | M89.729 |
| M89.8X1 | Other specified disorders of bone, shoulder |  |  | M89.8X1 |
| M89.8X2 | Other specified disorders of bone, upper arm |  |  | M89.8X2 |
| **Chondropathies** | | | | |
| M94.21 | Chondromalacia, shoulder | M94.212 | M94.211 | M94.219 |
| M94.8X1 | Other specified disorders of cartilage, shoulder |  |  | M94.8X1 |
| M94.8X2 | Other specified disorders of cartilage, upper arm |  |  | M94.8X2 |
| **Other disorders of the musculoskeletal system and connective tissue** | | | | |
| M95.8 | Other specified acquired deformities of musculoskeletal system |  |  | M95.8 |
| M95.9 | Acquired deformity of musculoskeletal system, unspecified |  |  | M95.9 |

**Table S1.3. Shoulder Symptom ICD-10-CM Codes (ICD-10 Code Group C)**

|  |  | **Laterality** | | |
| --- | --- | --- | --- | --- |
| **Code** | **Diagnosis** | **Left** | **Right** | **Unspecified** |
| M25.51 | Pain in shoulder | M25.512 | M25.511 | M25.519 |
| M25.61 | Stiffness of shoulder, not elsewhere classified | M25.612 | M25.611 | M25.619 |
| M79.6 | Pain in arm | M79.602 | M79.601 | M79.609 |
| M79.62 | Pain in upper arm | M79.622 | M79.621 | M79.629 |

**Table S1.4. Shoulder Injury Codes from ICD-10-CM Chapter 19: Injury, Poisoning and Certain Other Consequences of External Causes (ICD-10 Code Group D)**

|  |  | **Laterality** | | |  |
| --- | --- | --- | --- | --- | --- |
| **Code** | **Diagnosis** | **Left** | **Right** | **Unspecified** |  |
| **S40** | **Superficial injury of**  **shoulder and upper arm** | \| S40.012*, \| \| --- \| \| S40.022*, \| \| S40.212*, \| \| S40.222*, \| \| S40.242*, \| \| S40.252*, \| \| S40.262*, \| \| S40.272*, \| \| S40.812*, \| \| S40.822*, \| \| S40.842*, \| \| S40.852*, \| \| S40.862*, \| \| S40.872*, \| \| S40.912*, \| \| S40.922* \| | \| S40.011*, \| \| --- \| \| S40.021*, \| \| S40.211*, \| \| S40.221*, \| \| S40.241*, \| \| S40.251*, \| \| S40.261*, \| \| S40.271*, \| \| S40.811*, \| \| S40.821*, \| \| S40.841*, \| \| S40.851*, \| \| S40.861*, \| \| S40.871*, \| \| S40.911*, \| \| S40.921* \| | \| S40.019*, \| \| --- \| \| S40.029*, \| \| S40.219*, \| \| S40.229*, \| \| S40.249*, \| \| S40.259*, \| \| S40.269*, \| \| S40.279*, \| \| S40.819*, \| \| S40.829*, \| \| S40.849*, \| \| S40.859*, \| \| S40.869*, \| \| S40.879*, \| \| S40.919*, \| \| S40.929* \| | |
| **S41** | **Open wound of**  **shoulder and upper arm** | \| S41.002*, \| \| --- \| \| S41.012*, \| \| S41.022*, \| \| S41.032*, \| \| S41.042*, \| \| S41.052*, \| \| S41.102*, \| \| S41.112*, \| \| S41.122*, \| \| S41.132* \| \| S41.142*, \| \| S41.152* \| | \| S41.001*, \| \| --- \| \| S41.011*, \| \| S41.021*, \| \| S41.031*, \| \| S41.041*, \| \| S41.051*, \| \| S41.101*, \| \| S41.111*, \| \| S41.121*, \| \| S41.131* \| \| S41.141*, \| \| S41.151* \| | \| S41.009*, \| \| --- \| \| S41.019*, \| \| S41.029*, \| \| S41.039*, \| \| S41.049*, \| \| S41.059*, \| \| S41.109*, \| \| S41.119*, \| \| S41.129*, \| \| S41.139* \| \| S41.149*, \| \| S41.159* \| |  |
| **S42** | **Fracture of**  **shoulder and upper arm** |  |  |  |  |
| **S42.0** | **Fracture of**  **clavicle** | \| S42.002*, \| \| --- \| \| S42.012*, \| \| S42.022*, \| \| S42.032*, \| \| S42.015*, \| \| S42.015*, \| \| S42.018*, \| \| S42.018*, \| \| S42.034*, \| \| S42.035* \| | \| S42.001*, \| \| --- \| \| S42.011*, \| \| S42.021*, \| \| S42.031*, \| \| S42.014*, \| \| S42.014*, \| \| S42.017*, \| \| S42.017*, \| \| S42.024*, \| \| S42.034* \| | \| S42.009*, \| \| --- \| \| S42.013*, \| \| S42.016*, \| \| S42.019*, \| \| S42.023*, \| \| S42.029*, \| \| S42.026*, \| \| S42.039*, \| \| S42.033*, \| \| S42.036* \| |  |
| **S42.1** | **Fracture of**  **scapula** | \| S42.102*, \| \| --- \| \| S42.112*, \| \| S42.115*, \| \| S42.122*, \| \| S42.125*, \| \| S42.132*, \| \| S42.135*, \| \| S42.142*, \| \| S42.145*, \| \| S42.152*, \| \| S42.155*, \| \| S42.192* \| | \| S42.101*, \| \| --- \| \| S42.111*, \| \| S42.114*, \| \| S42.121*, \| \| S42.124*, \| \| S42.131*, \| \| S42.134*, \| \| S42.141*, \| \| S42.144*, \| \| S42.151*, \| \| S42.154*, \| \| S42.191* \| | \| S42.109*, \| \| --- \| \| S42.113*, \| \| S42.116*, \| \| S42.123*, \| \| S42.126*, \| \| S42.133*, \| \| S42.136*, \| \| S42.143*, \| \| S42.146*, \| \| S42.153*, \| \| S42.156*, \| \| S42.199* \| |  |
| **S42.2** | **Fracture of**  **upper end of humerus** | \| S42.202*, \| \| --- \| \| S42.212*, \| \| S42.215*, \| \| S42.222*, \| \| S42.225*, \| \| S42.232*, \| \| S42.242*, \| \| S42.252*, \| \| S42.255*, \| \| S42.262*, \| \| S42.265*, \| \| S42.272*, \| \| S42.292*, \| \| S42.295* \| | \| S42.201*, \| \| --- \| \| S42.211*, \| \| S42.214*, \| \| S42.221*, \| \| S42.224*, \| \| S42.231*, \| \| S42.241*, \| \| S42.251*, \| \| S42.254*, \| \| S42.261*, \| \| S42.264*, \| \| S42.271*, \| \| S42.291*, \| \| S42.294* \| | \| S42.209*, \| \| --- \| \| S42.213*, \| \| S42.216*, \| \| S42.223*, \| \| S42.226*, \| \| S42.239*, \| \| S42.249*, \| \| S42.253*, \| \| S42.256*, \| \| S42.263*, \| \| S42.266*, \| \| S42.279*, \| \| S42.293*, \| \| S42.296* \| |  |
| **S42.3** | **Fracture of**  **shaft of humerus** | \| S42.302*, \| \| --- \| \| S42.312*, \| \| S42.322*, \| \| S42.325*, \| \| S42.332*, \| \| S42.335*, \| \| S42.342*, \| \| S42.345*, \| \| S42.352*, \| \| S42.355*, \| \| S42.362*, \| \| S42.365*, \| \| S42.392* \| | \| S42.301*, \| \| --- \| \| S42.311*, \| \| S42.321*, \| \| S42.324*, \| \| S42.331*, \| \| S42.334*, \| \| S42.341*, \| \| S42.344*, \| \| S42.351*, \| \| S42.354*, \| \| S42.361*, \| \| S42.364*, \| \| S42.391* \| | \| S42.309*, \| \| --- \| \| S42.319*, \| \| S42.323*, \| \| S42.326*, \| \| S42.333*, \| \| S42.336*, \| \| S42.343*, \| \| S42.346*, \| \| S42.353*, \| \| S42.356*, \| \| S42.363*, \| \| S42.366*, \| \| S42.399* \| |  |
| **S42.4** | **Fracture of**  **lower end of humerus** | \| S42.402*, \| \| --- \| \| S42.412*, \| \| S42.415*, \| \| S42.422*, \| \| S42.425*, \| \| S42.432*, \| \| S42.435*, \| \| S42.442*, \| \| S42.445*, \| \| S42.448*, \| \| S42.452*, \| \| S42.455*, \| \| S42.462*, \| \| S42.465*, \| \| S42.472*, \| \| S42.475*, \| \| S42.482*, \| \| S42.492*, \| \| S42.495* \| | \| S42.401*, \| \| --- \| \| S42.411*, \| \| S42.414*, \| \| S42.421*, \| \| S42.424*, \| \| S42.431*, \| \| S42.434*, \| \| S42.441*, \| \| S42.444*, \| \| S42.447*, \| \| S42.451*, \| \| S42.454*, \| \| S42.461*, \| \| S42.464*, \| \| S42.471*, \| \| S42.474*, \| \| S42.481*, \| \| S42.491*, \| \| S42.494* \| | \| S43.409*, \| \| --- \| \| S43.413*, \| \| S43.416*, \| \| S43.423*, \| \| S43.426*, \| \| S43.433*, \| \| S43.436*, \| \| S43.443*, \| \| S43.446*, \| \| S43.449*, \| \| S43.453*, \| \| S43.456*, \| \| S43.463*, \| \| S43.466*, \| \| S43.473*, \| \| S43.476*, \| \| S43.489*, \| \| S43.493*, \| \| S43.496* \| |  |
| **S42.9** | **Fracture of shoulder girdle part unspecified** | S42.92X* | S42.91X* | S42.90X* | |
| **S43** | **Dislocation and sprain of joints and**  **ligaments of shoulder girdle** | \| S43.002*, \| \| --- \| \| S43.005*, \| \| S43.012*, \| \| S43.015*, \| \| S43.022*, \| \| S43.025*, \| \| S43.032*, \| \| S43.035*, \| \| S43.082*, \| \| S43.085*, \| \| S43.102*, \| \| S43.112*, \| \| S43.122*, \| \| S43.132*, \| \| S43.152*, \| \| S43.202*, \| \| S43.205*, \| \| S43.302*, \| \| S43.305*, \| \| S43.312*, \| \| S43.315*, \| \| S43.392*, \| \| S43.395*, \| \| S43.402*, \| \| S43.412*, \| \| S43.422*, \| \| S43.432*, \| \| S43.492*, \| \| S43.52X*, \| \| S43.62X*, \| \| S43.82X*, \| \| S43.92X* \| | \| S43.001*, \| \| --- \| \| S43.004*, \| \| S43.011*, \| \| S43.014*, \| \| S43.021*, \| \| S43.024*, \| \| S43.031*, \| \| S43.034*, \| \| S43.081*, \| \| S43.084*, \| \| S43.101*, \| \| S43.111*, \| \| S43.121*, \| \| S43.131*, \| \| S43.151*, \| \| S43.201*, \| \| S43.204*, \| \| S43.301*, \| \| S43.304*, \| \| S43.311*, \| \| S43.314*, \| \| S43.391*, \| \| S43.394*, \| \| S43.401*, \| \| S43.411*, \| \| S43.421*, \| \| S43.431*, \| \| S43.491*, \| \| S43.51X*, \| \| S43.61X*, \| \| S43.81X*, \| \| S43.91X* \| | \| S43.003*, \| \| --- \| \| S43.006*, \| \| S43.013*, \| \| S43.016*, \| \| S43.023*, \| \| S43.026*, \| \| S43.033*, \| \| S43.036*, \| \| S43.083*, \| \| S43.086*, \| \| S43.109*, \| \| S43.119*, \| \| S43.129*, \| \| S43.139*, \| \| S43.159*, \| \| S43.203*, \| \| S43.206*, \| \| S43.303*, \| \| S43.306*, \| \| S43.313*, \| \| S43.316*, \| \| S43.393*, \| \| S43.396*, \| \| S43.409*, \| \| S43.419*, \| \| S43.429*, \| \| S43.439*, \| \| S43.499*, \| \| S43.50X*, \| \| S43.60X*, \| \| S43.80X*, \| \| S43.90X* \| |  |
| **S44** | **Injury of nerves at**  **shoulder and upper arm level** | S44.32X*, S44.8X2*, S44.92X* | S44.31X*, S44.8X1*, S44.91X* | S44.30X*, S44.8X9*, S44.90X* |  |
| **S45** | **Injury of blood vessels at**  **shoulder and upper arm level** | \| S45.002*, \| \| --- \| \| S45.012*, \| \| S45.092*, \| \| S45.202*, \| \| S45.212*, \| \| S45.292*, \| \| S45.802*, \| \| S45.812*, \| \| S45.892*, \| \| S45.902*, \| \| S45.912*, \| \| S45.992* \| | \| S45.001*, \| \| --- \| \| S45.011*, \| \| S45.091*, \| \| S45.201*, \| \| S45.211*, \| \| S45.291*, \| \| S45.801*, \| \| S45.811*, \| \| S45.891*, \| \| S45.901*, \| \| S45.911*, \| \| S45.991* \| | \| S45.009*, \| \| --- \| \| S45.019*, \| \| S45.099*, \| \| S45.209*, \| \| S45.219*, \| \| S45.299*, \| \| S45.809*, \| \| S45.819*, \| \| S45.899*, \| \| S45.909*, \| \| S45.919*, \| \| S45.999* \| |  |
| **S46** | **Injury of muscle fascia and tendon**  **at shoulder and upper arm level** | \| S46.002*, \| \| --- \| \| S46.012*, \| \| S46.022*, \| \| S46.092*, \| \| S46.802*, \| \| S46.812*, \| \| S46.822*, \| \| S46.892*, \| \| S46.902*, \| \| S46.912*, \| \| S46.922*, \| \| S46.982*, \| \| S46.992* \| | \| S46.001*, \| \| --- \| \| S46.011*, \| \| S46.021* \| \| S46.091*, \| \| S46.801*, \| \| S46.811*, \| \| S46.821*, \| \| S46.891*, \| \| S46.901*, \| \| S46.911*, \| \| S46.921*, \| \| S46.981*, \| \| S46.991* \| | \| S46.009*, \| \| --- \| \| S46.019*, \| \| S46.029* \| \| S46.099*, \| \| S46.809*, \| \| S46.819*, \| \| S46.829*, \| \| S46.899*, \| \| S46.909*, \| \| S46.919*, \| \| S46.929*, \| \| S46.989*, \| \| S46.999* \| |  |
| **S47** | **Crushing injury of**  **shoulder and upper arm** | S47.2XX* | S47.1XX* | S47.9XX* |  |
| **S48** | **Traumatic amputation of**  **shoulder and upper arm** | \| S48.012*, \| \| --- \| \| S48.022*, \| \| S48.112*, \| \| S48.122*, \| \| S48.912*, \| \| S48.922* \| | \| S48.011*, \| \| --- \| \| S48.021*, \| \| S48.111*, \| \| S48.121*, \| \| S48.911*, \| \| S48.921* \| | \| S48.019*, \| \| --- \| \| S48.029*, \| \| S48.119*, \| \| S48.129*, \| \| S48.919*, \| \| S48.929* \| |  |
| **S49** | **Other and unspecified injuries of**  **shoulder and upper arm** | \| S49.002*, \| \| --- \| \| S49.012*, \| \| S49.022*, \| \| S49.032*, \| \| S49.042*, \| \| S49.092*, \| \| S49.102*, \| \| S49.112*, \| \| S49.122*, \| \| S49.132*, \| \| S49.142*, \| \| S49.192*, \| \| S49.82X*, \| \| S49.92X* \| | \| S49.001*, \| \| --- \| \| S49.011*, \| \| S49.021*, \| \| S49.031*, \| \| S49.041*, \| \| S49.091*, \| \| S49.101*, \| \| S49.111*, \| \| S49.121*, \| \| S49.131*, \| \| S49.141*, \| \| S49.191*, \| \| S49.81X*, \| \| S49.91X* \| | \| S49.009*, \| \| --- \| \| S49.019*, \| \| S49.029*, \| \| S49.039*, \| \| S49.049*, \| \| S49.099*, \| \| S49.109*, \| \| S49.119*, \| \| S49.129*, \| \| S49.139*, \| \| S49.149*, \| \| S49.199*, \| \| S49.80X*, \| \| S49.90X* \| |  |
