# Supplementary material for: Identifying Cases of Shoulder Injury Related to Vaccine Administration (SIRVA) in the United States: Development and Validation of a Natural Language Processing Method
Source: JMIR Public Health Surveill. 2022 May 24;8(5):e30426. doi: 10.2196/30426 (PMC9175103; doi:10.2196/30426)
Supplement: Multimedia Appendix 2 [file publichealth_v8i5e30426_app2.docx]

**Appendix 2. Abstraction form**

**Identifying Cases of Shoulder Injury Related to Vaccine Administration (SIRVA) in the United States: Development and Validation of a Natural Language Processing Method**

Chengyi Zheng^1^, PhD, Jonathan Duffy^2^, MD, In-Lu Amy Liu^1^, MS, Lina S. Sy^1^, MPH, Ronald A. Navarro^3^, MD, Sunhea S. Kim^1^, MPH, Denison S. Ryan^1^, MPH, Wansu Chen^1^, PhD, Lei Qian^1^, PhD, Cheryl Mercado^1^, MPH, Steven J. Jacobsen^1^, MD, PhD

^1^ Department of Research & Evaluation, Kaiser Permanente Southern California, Pasadena, California, USA

^2^ Immunization Safety Office, Centers for Disease Control and Prevention, Atlanta, GA, USA

^3^ Kaiser Permanente South Bay Medical Center, Harbor City, California

**Corresponding Author:**

Chengyi Zheng, PhD

Department of Research and Evaluation, Kaiser Permanente Southern California

100 S Los Robles Ave, 2nd Floor,

Pasadena, CA 91101

United States

Phone: 1 626 986 8665

Email: Chengyi.X.Zheng@kp.org

**Abstraction form**

1. SCK Study ID (pre-populated)
2. VSD Study ID (pre-populated)

**A. BACKGROUND INFORMATION**

1. Abstractor initials
2. Abstraction date

**B. VACCINATION**

**We are interested in vaccines administered in 2016-2017.**

**The vaccine types and date are prepopulated.**

5-1). Vaccine type (pre-populated)

5-2).

5-3).

5-4).

1. Vaccination date (pre-populated)
2. Was there a vaccine given on this day? Yes

No

Unknown


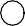

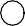

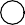


1. Are there clinic notes available for the Yes

vaccination visit? No


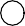

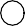


(If there is registry information but no clinic notes, select "No")

8-1). If yes, was vaccination documented in the chart Yes

notes? (e.g., nursing notes, etc.) Encounter notes available but no mention of vaccination


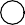

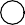


Documented in the immunization record only Mentioned in clinic notes but not noted as given - documented in the immunization record


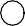

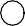


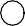
 Other

8-2). Please copy and paste the relevant statement for the vaccine(s) given in the chart notes.

8-3). Give the statement to specify Other.

1. Was the route of the administration for the Yes

vaccine intramuscular (IM)? No


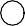

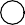

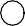


Unknown

1. Was the vaccine administered in the deltoid Yes

muscle (i.e., the upper arm)? No


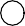

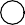

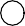


Unknown

1. Vaccination side from SCK data (pre-populated) Right Left Both

Unknown


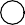

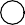

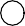

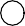


1. Vaccination side noted in the chart/registry Right Left Both

Unknown


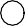

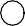

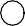

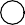


1. Shoulder of interest Right

Left


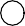

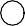

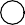

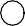


If the side from the chart/registry (right, left, or Both

both) does not agree with the prepopulated value Unknown above, then use the chart value. If no chart value

is available, then use the prepopulated value. Hereafter, this will be referred to as the "shoulder of interest" and will be populated in questions about the side of symptoms/injury.

1. The setting of vaccination Clinic/Doctor's Office Urgent Care

Emergency Room Hospital Inpatient Pharmacy

Work


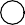

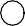

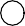

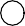

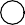

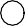

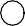

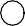

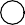


Flu vaccine clinic/booth

Other (specify)

Unknown

(Select the location that best describes where the patient was vaccinated. If the patient is a healthcare worker who received his/her vaccination at the place of employment, select "Work.")

14-1). Specify other location of vaccination.

1. Credentials of the vaccinator
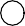
 LVN (Licensed Vocational Nurse)/LPN (Licensed Practical Nurse)

MA (Medical Assistant) MD/DO (Physician)


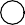

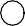

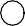

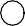

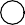

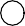

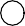

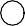

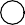


NP (Nurse Practitioner)

PA (Physician's Assistant) Pharmacist

Pharmacy Assistant

RN (Registered Nurse) Unknown

Other (specify)

15-1). Specify other credentials of the vaccinator.

14R. The setting of vaccination in RIGHT shoulder/arm Clinic/Doctor's Office Urgent Care

Emergency Room Hospital Inpatient Pharmacy

Work


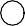

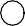

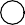

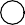

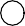

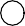

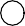

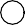

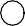


Flu vaccine clinic/booth

Other (specify)

Unknown

(Select the location that best describes where the patient was vaccinated. If the patient is a healthcare worker who received his/her vaccination at the place of employment, select "Work.")

14R-1). Specify other locations of vaccination in RIGHT shoulder/arm.

15R. Credentials of the vaccinator in RIGHT
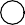
 LVN (Licensed Vocational Nurse)/LPN (Licensed

shoulder/arm Practical Nurse)

MA (Medical Assistant) MD/DO (Physician)


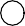

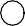

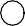

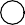

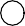

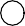

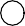

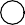

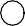


NP (Nurse Practitioner)

PA (Physician's Assistant) Pharmacist

Pharmacy Assistant

RN (Registered Nurse) Unknown

Other (specify)

15R-1). Specify other credentials of vaccinator in RIGHT shoulder/arm.

14L. The setting of vaccination in LEFT shoulder/arm Clinic/Doctor's Office Urgent Care


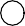

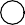

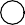

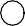

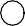

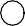

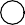

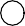

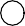


Emergency Room Hospital Inpatient Pharmacy

Work

Flu vaccine clinic/booth

Other (specify)

Unknown

(Select the location that best describes where the patient was vaccinated. If the patient is a healthcare worker who received his/her vaccination at the place of employment, select "Work.")

14L-1). Specify other locations of vaccination in LEFT shoulder/arm.

15L. Credentials of the vaccinator in LEFT
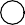
 LVN (Licensed Vocational Nurse)/LPN (Licensed

shoulder/arm Practical Nurse)

MA (Medical Assistant) MD/DO (Physician)


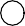

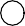

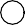

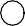

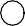

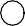

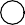

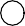

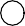


NP (Nurse Practitioner)

PA (Physician's Assistant) Pharmacist

Pharmacy Assistant

RN (Registered Nurse) Unknown

Other (specify)

15L-1). Specify other credentials of vaccinator in LEFT shoulder/arm.

1. Were any problems with the vaccine injection Yes

noted (e.g., administration error)? No


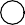

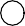


16-1). If yes, specify errors noted.

1. Did the patient have any post-vaccination Yes

shoulder or arm (same as vaccinated) complaints No noted at this visit?


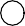

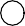


1. Does the note mention any previous shoulder Yes

symptoms? No


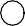

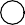


18-1). Side of previous shoulder symptoms Right Left

Both

Unknown


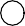

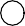

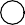

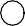


18-2). Please copy and paste the relevant statement

for previous shoulder symptoms and side mentioned from the chart notes.

**C. MEDICAL ENCOUNTERS ON THE SAME DAY AS VACCINATION**

1. Were there any other provider visits on the same Yes


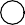

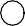

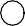


calendar day as vaccination? No

Unknown

Please only include encounters with patient/provider interaction, providers can include physicians, nurses, physical therapists, and other allied health workers.

1. Type of encounter
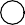
 Outpatient (primary care and specialty care office visit)

Urgent care


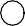


Emergency room Hospital inpatient

Phone call

Email

Other (specify)

20-1). Specify other.

1. Provider type Primary Care

ER/Urgent Care/Inpatient Shoulder specialist

Non-shoulder specialist

Other (specify)

21-1). Specify other.

1. Were any shoulder symptoms noted at this visit? Yes

No

22-1). On which side were the shoulder symptoms Right

noted? Left

Both Unknown

1. List any shoulder or arm diagnoses made at this

visit. (Leave this blank if no diagnosis is given).

1. When did the shoulder or arm symptoms start?

Please be as specific as possible. If a date is (If the duration of symptoms is not discussed, leave given, give the date. this blank.)

- If a duration is given (e.g., "two weeks ago"), count backward that amount of time from the visit date and use that as the start date, even if the duration given was inexact (e.g., "about two weeks ago").
- If a range is given (e.g., "one to two weeks ago"), give the earliest possible start date (in that case, two weeks prior to the appointment).
- Use the calendar box to select a date and write the exact statement regarding the start date or symptom duration in the text box.

1. Is this date exact or an estimation? Exact Estimate

(Select "estimate" if symptom duration/onset date was not discussed.)

1. Is this symptom onset date before OR after Before

vaccination? After

Same

26-1). If the symptom onset date is the same as the Before

vaccination date, is symptom onset before or after the After vaccination?

1. Give the statement regarding the description of the symptoms and start date or symptom duration as

it is in the chart. If the duration is not discussed, enter that here as well.

1. Was the shoulder or arm diagnosis attributed to Yes

the vaccination by the provider? No

Not stated

1. Please copy and paste the relevant statement for any cause of the shoulder symptoms from the chart

note AND save the full visit note (unredacted) to S: drive as part of a case packet.

24R. When did the RIGHT shoulder or arm symptoms start?

(If the duration of symptoms is not discussed, leave

Please be as specific as possible. If a date is this blank.) given, give the date.

- If a duration is given (e.g., "two weeks ago"), count backward that amount of time from the visit date and use that as the start date, even if the duration given was inexact (e.g., "about two weeks ago").
- If a range is given (e.g., "one to two weeks ago"), give the earliest possible start date (in that case, two weeks prior to the appointment).
- Use the calendar box to select a date and write the exact statement regarding the start date or symptom duration in the text box.

25R. Is this date exact or an estimation? Exact Estimate

(Select "estimate" if symptom duration/onset date was not discussed.)

26R. Is the RIGHT shoulder symptom onset date before OR Before after vaccination? After

Same

26R-1). If the RIGHT shoulder symptom onset date is Before

the same as the vaccination date, is symptom onset After before or after the vaccination?

27R. Give the statement regarding the description of the RIGHT shoulder symptoms and start date or RIGHT

shoulder symptom duration as it is in the chart. If the duration is not discussed, enter that here as well.

28R. Was the RIGHT shoulder or arm diagnosis Yes

attributed to the vaccination by the provider? No

Not stated

29R. Please copy and paste the relevant statement for any cause of the RIGHT shoulder symptoms from the

chart note AND save the full visit note (unredacted) to S: drive as part of a case packet.

24L. When did the LEFT shoulder or arm symptoms start?

(If the duration of symptoms is not discussed, leave

Please be as specific as possible. If a date is this blank.) given, give the date.

- If a duration is given (e.g., "two weeks ago"), count backward that amount of time from the visit date and use that as the start date, even if the duration given was inexact (e.g., "about two weeks ago").
- If a range is given (e.g., "one to two weeks ago"), gives the earliest possible start date (in that case, two weeks prior to the appointment).
- Use the calendar box to select a date and write the exact statement regarding the start date or symptom duration in the text box.

25L. Is this date exact or an estimation? Exact Estimate

(Select "estimate" if symptom duration/onset date was not discussed.)

26L. Is the LEFT shoulder symptom onset date before OR Before after vaccination? After

Same

26L-1). If the LEFT shoulder symptom onset date is Before

the same as the vaccination date, is LEFT shoulder After symptom onset before or after the vaccination?

27L. Give the statement regarding the description of the LEFT shoulder symptoms and start date or LEFT

shoulder symptom duration as it is in the chart. If the duration is not discussed, enter that here as well.

28L. Was the LEFT shoulder or arm diagnosis Yes

attributed to the vaccination by the provider? No

Not stated

29L. Please copy and paste the relevant statement for any cause of the LEFT shoulder symptoms from the

chart note AND save the full visit note (unredacted) to S: drive as part of a case packet.

**D. PRE-EXISTING SHOULDER CONDITIONS DOCUMENTED PRIOR TO VACCINATION DATE**

**Working backward from the date of vaccination to 6 months prior to vaccination, look for any visits for shoulder or upper arm problems, symptoms, or injuries. Do not worry about any injuries or symptoms to the elbow, forearm, wrist, or hand.**

1. Any prior shoulder/upper arm symptoms found? Yes

No

30-1). On which side were the shoulder symptoms Right

noted? Left

Both Unknown

1. Visit date of most recent visit for

shoulder/upper arm symptoms prior to vaccination

1. Provider type Primary Care

ER/Urgent Care/Inpatient Shoulder specialist

Non-shoulder specialist

Other (specify)

32-1). Specify other providers.

1. Was there a previous diagnosis of the following Yes

shoulder injuries in the above shoulder in 6 months No prior to vaccination?

- Adhesive capsulitis/ frozen shoulder
- Bone erosion
- Bursitis
- Humerus fractures
- Impingement
- Left shoulder joint pain
- Osteitis
- Osteolysis
- Osteonecrosis
- Periosteal reactions
- Pseudoseptic arthritis
- Right shoulder joint pain
- Rotator cuff syndrome
- Shoulder joint effusion
- Synovitis/ tenosynovitis
- Tendinitis/ tendinosis/ tendonitis/ tendinopathy
- Torn rotator cuff

1. Was there a previous shoulder/upper arm diagnosis Yes other than the above shoulder injuries (Q33) in the No above shoulder?

34-1). Specify other shoulder/upper arm diagnoses

(list all shoulder/upper arm diagnoses given during the 6 months prior to vaccination).

31R. Visit date of most recent visit for RIGHT

shoulder/upper arm symptoms prior to vaccination

32R. Provider type Primary Care

ER/Urgent Care/Inpatient Shoulder specialist

Non-shoulder specialist

Other (specify)

32R-1). Specify other provider.

33R. Was there a previous diagnosis of the following Yes

shoulder injuries in the RIGHT shoulder in 6 months No prior to vaccination?

- Adhesive capsulitis/ frozen shoulder
- Bone erosion
- Bursitis
- Humerus fractures
- Impingement
- Left shoulder joint pain
- Osteitis
- Osteolysis
- Osteonecrosis
- Periosteal reactions
- Pseudoseptic arthritis
- Right shoulder joint pain
- Rotator cuff syndrome
- Shoulder joint effusion
- Synovitis/ tenosynovitis
- Tendinitis/ tendinosis/ tendonitis/ tendinopathy
- Torn rotator cuff

34R. Was there a previous shoulder/upper arm Yes

diagnosis other than the above shoulder injuries No (Q33R) in the RIGHT shoulder?

34R-1). Specify other RIGHT shoulder/upper arm

diagnoses (list all shoulder/upper arm diagnoses given during the 6 months prior to vaccination).

31L. Visit date of most recent visit for LEFT

shoulder/upper arm symptoms prior to vaccination

32L. Provider type Primary Care

ER/Urgent Care/Inpatient Shoulder specialist

Non-shoulder specialist

Other (specify)

32L-1). Specify other providers.

33L. Was there a previous diagnosis of the following Yes

shoulder injuries in the LEFT shoulder in 6 months No prior to vaccination?

- Adhesive capsulitis/ frozen shoulder
- Bone erosion
- Bursitis
- Humerus fractures
- Impingement
- Left shoulder joint pain
- Osteitis
- Osteolysis
- Osteonecrosis
- Periosteal reactions
- Pseudoseptic arthritis
- Right shoulder joint pain
- Rotator cuff syndrome
- Shoulder joint effusion
- Synovitis/ tenosynovitis
- Tendinitis/ tendinosis/ tendonitis/ tendinopathy
- Torn rotator cuff

34L. Was there a previous LEFT shoulder/upper arm Yes

diagnosis other than the above shoulder injuries No (Q33L) in the above shoulder?

34L-1). Specify other LEFT shoulder/upper arm

diagnoses (list all shoulder/upper arm diagnoses given during the 6 months prior to vaccination).

**E. NEXT MEDICAL ENCOUNTER AFTER VACCINATION**

**We are interested in the first medical encounter that happened on a date other than the vaccination date, within 30 days of vaccination, even if it is not related to the shoulder/arm or vaccination.**

1. Is there an encounter within 30 days of Yes

vaccination? No

1. Date of next medical encounter
2. Type of encounter Outpatient (primary care and specialty care office visit)

Urgent care

Emergency room Hospital inpatient

Phone call

Email

Other (specify)

37-1). Specify other.

1. Provider type Primary Care

ER/Urgent Care/Inpatient Shoulder specialist

Non-shoulder specialist

Other (specify)

38-1). If other, specify.

1. What was the chief complaint of this visit?
2. Were any shoulder/upper arm symptoms documented as Yes part of this encounter? No

40-1). On which side did the patient complain of Right

shoulder/upper arm symptoms? Left

Both Unknown

**F. FIRST SHOULDER/UPPER ARM VISIT WITHIN 30 DAYS POST-VACCINATION**

**Look for the first clinic visit for shoulder/upper arm symptoms, within 30 days of vaccination. If the visit is clearly NOT for the shoulder of interest, go to the first visit that is for the shoulder of interest/both shoulders/laterality is unclear.**

**If the answer to the previous question (Q40. Did the patient complain of shoulder/upper arm symptoms as part of this encounter?) is Yes, answer these questions based on that visit. If the answer was No, look for the next clinic visit that involves shoulder/upper arm symptoms and answer the questions based on that visit.**

1. Is there a shoulder/upper arm visit within 30 Yes

days of the vaccination date? No

1. Date of the first shoulder/upper arm visit.

(If there are no shoulder visits please enter '09/09/9999'.)

1. Type of encounter Outpatient (primary care and specialty care office visit)

Urgent care

Emergency room

Hospital inpatient

Other (specify)

43-1). Specify other.

1. Type of provider Orthopedics

Rheumatologist

Physical Medicine & Rehabilitation

Sports Medicine

Primary Care Physician (e.g., internal medicine, family medicine, pediatrics)

Physical Therapy Occupational Therapy

Other (specify)

44-1). Specify other provider type.

1. Which side has the symptoms? Right Left

Both

Unknown

1. When did the shoulder or arm symptoms start?

Please be as specific as possible. If a date is given, give the (Leave blank if symptom duration or onset date was, date. If a duration is given (e.g., "two weeks ago") not discussed at this visit.)

count backwards that amount of time from the visit date and use that as the start date, even if the duration given was inexact (e.g., "about two weeks ago"). If a range is given (e.g., "one to two weeks ago") give the earliest possible start date (in that case, two weeks prior to the appointment). Use the calendar box to select a date and write the exact statement regarding the start date or symptom duration in the text box.

1. Is this date exact or an estimation? Exact Estimate

(Select "estimate" if the duration of symptoms was not discussed.)

1. Is this pre-existing condition? Yes

No

Possible Unknown

1. Were the pre-existing shoulder/arm symptoms noted to Yes recur or worsen following vaccination? No

Possible

If no, please STOP here and END abstraction. Unknown

1. Did the recurrence or worsening of symptoms occur Yes within 30 days of vaccination? No

Unknown

If no, please STOP here and END abstraction.

1. Give the statement regarding the description of the symptoms, start date or symptom duration as it

is in the chart. If duration is not discussed, enter that here as well.

Please save the full visit note (unredacted) to S: drive as part of a case packet.

1. Check all the symptoms that were reported Atrophy Impingement Numbness Pain/Soreness

Radiating pain (from a non-shoulder location) Reduced range of motion (ROM)

Stiffness

Swelling

Tingling

Weakness

Other shoulder symptoms (specify)

52-1). Specify other.

1. Was a specific injury or action, other than Yes

vaccination, mentioned as the cause of the No shoulder/upper arm symptoms?

1. Were the shoulder/upper arm symptoms attributed to Yes the vaccine by the patient or provider? No
2. Please copy and paste the relevant statement from the chart note for any cause of the symptoms AND

save the full visit note (unredacted) to S: drive as part of a case packet.

1. Was vaccination mentioned at all in the visit Yes

note? No

1. Was the vaccine type specified in the statement? Yes

No

57-1). If the above question is "Yes", select the Influenza

vaccine type from the following list. TDAP Pneumococcal Hepatitis A or B HPV

Meningococcal Other (specify)

57-1a). Specify other.

57-2). Does the vaccine type state match to the one Yes

listed in Q5? No

1. Were any symptoms documented described as being Induration/Hard mass related to a vaccine injection? Select as many as apply. Erythema/Swelling/Redness

Pain/Soreness

Rash

Other (specify)

58-1). Specify other.

1. Specify positive/abnormal physical exam findings pertaining to the above shoulder/upper arm.
2. Was there a diagnosis of the following shoulder Adhesive capsulitis/ frozen shoulder injuries in the above shoulder at this visit? Please Bone erosion

select as many diagnoses given at this visit. Bursitis Impingement Osteitis Osteolysis Osteonecrosis

Periosteal reactions Pseudoseptic arthritis Shoulder joint effusion Synovitis/ tenosynovitis

Tendinitis/ tendinosis/ tendonitis/ tendinopathy Torn rotator cuff

Rotator cuff syndrome

Right shoulder joint pain

Left shoulder joint pain

Humerus fractures

Other

No shoulder injury diagnosis

60-1). Specify other shoulder/upper arm diagnoses (If

there is no Other diagnosis, please leave blank.)

46R. When did the RIGHT shoulder or arm symptoms start?

(Leave blank if symptom duration or onset date was

Please be as specific as possible. If a date is given, give the not discussed at this visit.) date. If a duration is given (e.g., "two weeks ago")

count backward that amount of time from the visit date and use that as the start date, even if the duration given was inexact (e.g., "about two weeks ago"). If a range is given (e.g., "one to two weeks ago") give the earliest possible start date (in that case, two weeks prior to the

appointment). Use the calendar box to select a date and write the exact statement regarding the start date or symptom duration in the text box.

47R. Is this date exact or an estimation? Exact Estimate

(Select "estimate" if the duration of symptoms was not discussed.)

48R. Is this pre-existing condition? Yes

No

Possible Unknown

49R. Were the pre-existing RIGHT shoulder/arm Yes

symptoms noted to recur or worsen following No

vaccination? Possible

Unknown

If no, please STOP here and END abstraction.

50R. Did the recurrence or worsening of RIGHT Yes

shoulder symptoms occur within 30 days of vaccination? No

Unknown

If no, please STOP here and END abstraction.

51R. Give the statement regarding the description of the RIGHT shoulder symptoms, start date or symptom

duration as it is in the chart. If duration is not discussed, enter that here as well.

Please save the full visit note (unredacted) to S: drive as part of a case packet.

52R. Check all the RIGHT shoulder symptoms that were Atrophy reported Impingement

Numbness

Pain/Soreness

Radiating pain (from a non-shoulder location) Reduced range of motion (ROM)

Stiffness

Swelling

Tingling

Weakness

Other shoulder symptoms (specify)

52R-1). Specify other.

53R. Was a specific injury or action, other than Yes

vaccination, mentioned as the cause of the RIGHT No shoulder/upper arm symptoms?

54R. Were the RIGHT shoulder/upper arm symptoms Yes

attributed to the vaccine by the patient or provider? No

55R. Please copy and paste the relevant statement from the chart note for any cause of the RIGHT

shoulder symptoms AND save the full visit note (unredacted) to S: drive as part of a case packet.

56R. Was vaccination mentioned at all in the visit note? Yes

No

57R. Was the vaccine type specified in the statement? Yes

No

57R-1). If the above question is "Yes", select the Influenza

vaccine type from the following list. TDAP Pneumococcal Hepatitis A or B HPV

Meningococcal Other (specify)

57R-1a). Specify other.

57R-2). Does the vaccine type stated match to the one Yes listed in Q5? No

58R. Were any RIGHT shoulder symptoms documented Induration/Hard mass described as being related to a vaccine injection? Erythema/Swelling/Redness Select as many as apply. Pain/Soreness

Rash

Other (specify)

58R-1). Specify other.

59R. Specify positive/abnormal physical exam findings pertaining to the RIGHT shoulder/upper arm.

60R. Was there a diagnosis of the following shoulder Adhesive capsulitis/ frozen shoulder injuries in the RIGHT shoulder at this visit? Please Bone erosion

select as many diagnoses are given at this visit. Bursitis Impingement Osteitis Osteolysis Osteonecrosis

Periosteal reactions Pseudoseptic arthritis Shoulder joint effusion Synovitis/ tenosynovitis

Tendinitis/ tendinosis/ tendonitis/ tendinopathy Torn rotator cuff

Rotator cuff syndrome

Right shoulder joint pain

Left shoulder joint pain

Humerus fractures

Other

No shoulder injury diagnosis

60R-1). Specify other RIGHT shoulder/upper arm

diagnoses (If there are no Other diagnosis, please leave blank.)

46L. When did the LEFT shoulder or arm symptoms start?

(Leave blank if symptom duration or onset date was

Please be as specific as possible. If a date is given, give the not discussed at this visit.) date. If a duration is given (e.g., "two weeks ago")

count backward that amount of time from the visit date and use that as the start date, even if the duration given was inexact (e.g., "about two weeks ago"). If a range is given (e.g., "one to two weeks ago") give the earliest possible start date (in that case, two weeks prior to the

appointment). Use the calendar box to select a date and write the exact statement regarding the start date or symptom duration in the text box.

47L. Is this date exact or an estimation? Exact Estimate

(Select "estimate" if the duration of symptoms was not discussed.)

48L. Is this pre-existing condition? Yes

No

Possible Unknown

49L. Were the pre-existing LEFT shoulder/arm symptoms Yes noted to recur or worsen following vaccination? No

Possible

If no, please STOP here and END abstraction. Unknown

50L. Did the recurrence or worsening of LEFT shoulder Yes symptoms occur within 30 days of vaccination? No

Unknown

If no, please STOP here and END abstraction.

51L. Give the statement regarding the description of the LEFT shoulder symptoms, start date or symptom

duration as it is in the chart. If duration is not discussed, enter that here as well.

Please save the full visit note (unredacted) to S: drive as part of a case packet.

52L. Check all the LEFT shoulder symptoms that were Atrophy reported Impingement

Numbness

Pain/Soreness

Radiating pain (from a non-shoulder location) Reduced range of motion (ROM)

Stiffness

Swelling

Tingling

Weakness

Other shoulder symptoms (specify)

52L-1). Specify other.

53L. Was a specific injury or action, other than Yes

vaccination, mentioned as the cause of the LEFT No shoulder/upper arm symptoms?

54L. Were the LEFT shoulder/upper arm symptoms Yes

attributed to the vaccine by the patient or provider? No

55L. Please copy and paste the relevant statement from the chart note for any cause of the LEFT

shoulder symptoms AND save the full visit note (unredacted) to S: drive as part of a case packet.

56L. Was vaccination mentioned at all in the visit note? Yes

No

57L. Was the vaccine type specified in the statement? Yes

No

57L-1). If the above question is "Yes", select the Influenza

vaccine type from the following list. TDAP Pneumococcal Hepatitis A or B HPV

Meningococcal Other (specify)

57L-1a). Specify other.

57L-2). Does the vaccine type stated match the one Yes listed in Q5? No

58L. Were any LEFT shoulder symptoms documented Induration/Hard mass described as being related to a vaccine injection? Erythema/Swelling/Redness Select as many as apply. Pain/Soreness

Rash

Other (specify)

58L-1). Specify other.

59L. Specify positive/abnormal physical exam findings pertaining to the LEFT shoulder/upper arm.

60L. Was there a diagnosis of the following shoulder Adhesive capsulitis/ frozen shoulder injuries in the LEFT shoulder at this visit? Please Bone erosion

select as many diagnoses given at this visit. Bursitis Impingement Osteitis Osteolysis Osteonecrosis

Periosteal reactions Pseudoseptic arthritis Shoulder joint effusion Synovitis/ tenosynovitis

Tendinitis/ tendinosis/ tendonitis/ tendinopathy Torn rotator cuff

Rotator cuff syndrome

Right shoulder joint pain

Left shoulder joint pain Humerus fractures

Other

No shoulder injury diagnosis

60L-1). Specify other LEFT shoulder/upper arm

diagnoses (If there are no Other diagnosis, please leave blank.)

61-1). List all other diagnoses below given at this

appointment, even if they don't pertain to the shoulder.

61-2).

61-3).

61-4).

61-5).

61-6).

61-7).

61-8).

61-9).

61-10).

61-11). Please include all other diagnoses if more than ten.

**G. FIRST SHOULDER/UPPER ARM VISIT WITHIN 31-180 DAYS POST-VACCINATION**

**Look for the first clinic visit for shoulder/upper arm symptoms, within 31-180 days of vaccination. If the visit is clearly NOT for the shoulder of interest, go to the first visit that is for the shoulder of interest/both shoulders/laterality is unclear.**

1. Is there a visit related to the shoulder/arm of Yes

interest within 31-180 days following vaccination? No

If no, go to Section H.

1. Date of visit:
2. Type of encounter: Outpatient (primary care and specialty care office visit)

Urgent care

Emergency room Hospital inpatient

Other (specify)

64-1). Specify other.

1. Type of provider: Orthopedics

Rheumatologist

Physical Medicine & Rehabilitation

Sports Medicine

Primary Care Physician (e.g., internal medicine, family medicine, pediatrics)

Physical Therapy Occupational Therapy

Other (specify)

65-1). Specify other provider types.

1. Which shoulder was discussed at the encounter? Right Left

Both

Unknown

1. Did the patient note that his/her symptoms had Resolved

resolved/relieved? Improved

No resolution/improvement

Other (e.g., some symptoms improved but other symptoms still present)

Unknown

1. Symptoms that the patient is still experiencing: Atrophy Impingement Numbness Pain/Soreness

Radiating pain (from non-shoulder location) Reduced range of motion (ROM)

Stiffness

Swelling

Tingling

Weakness

Other shoulder symptoms (specify)

68-1). Specify other.

1. Was a specific injury or action, other than Yes

vaccination, mentioned as the cause of the No shoulder/upper arm symptoms?

1. Were the shoulder/upper arm symptoms attributed to Yes the vaccine by the patient or provider? No
2. Please copy and paste the relevant statement from the chart note for any cause of the symptoms AND

save the full visit note (unredacted) to S: drive as part of a case packet.

1. Was vaccination mentioned at all in the visit Yes

note? No

1. Was the vaccine type specified in the statement? Yes

No

73-1). If the above question is "Yes", select the Influenza

vaccine type from the following list. TDAP Pneumococcal Hepatitis A or B HPV

Meningococcal Other (specify)

73-1a). Specify other.

73-2). Does the vaccine type state match the one Yes

listed in Q5? No

67R. Did the patient note that his/her RIGHT shoulder Resolved symptoms had resolved/relieved? Improved

No resolution/improvement

Other (e.g., some symptoms improved but other symptoms still present)

Unknown

68R. RIGHT shoulder symptoms that the patient is Atrophy

still experiencing: Impingement

Numbness

Pain/Soreness

Radiating pain (from non-shoulder location) Reduced range of motion (ROM)

Stiffness

Swelling

Tingling

Weakness

Other shoulder symptoms (specify)

68R-1). Specify other.

67L. Did the patient note that his/her LEFT shoulder Resolved

symptoms had resolved/relieved? Improved

No resolution/improvement

Other (e.g., some symptoms improved but other symptoms still present)

Unknown

68L. LEFT shoulder symptoms that the patient is still Atrophy

experiencing: Impingement

Numbness

Pain/Soreness

Radiating pain (from non-shoulder location) Reduced range of motion (ROM)

Stiffness

Swelling

Tingling

Weakness

Other shoulder symptoms (specify)

68L-1). Specify other.

74-1). List all diagnoses given at this appointment,

even if they don't pertain to the shoulder.

74-2).

74-3).

74-4).

74-5).

74-6).

74-7).

74-8).

74-9).

74-10).

74-11). Please include all other diagnoses if more than ten.

**H. Case Definition**

**A SIRVA case is a shoulder injury occurring in the same arm in which a vaccine was injected within the first 7 days following vaccination and lasting more than 30 days following vaccination, with vaccination as one of the possible causes of the shoulder injury.**

**Please use all available records in +/- 6 months from the index date to answer the questions in this section.**

75. In your review, which shoulder/arm was identified Right

with symptoms after vaccination? Left

Both

Unknown

75R. Did a true RIGHT shoulder injury occur? (from Shoulder injury diagnosis code AND

the following) physician/patient mention of shoulder symptoms

Shoulder injury diagnosis code with no mention of

- Adhesive capsulitis/ frozen shoulder shoulder symptoms by physician/patient
- Bone erosion Shoulder symptoms mentioned by physician/patient
- Bursitis but no shoulder injury diagnosis code
- Humerus fractures No shoulder injury diagnosis code or
- Impingement physician/patient mention of shoulder symptoms
- Left shoulder joint pain
- Osteitis
- Osteolysis
- Osteonecrosis
- Periosteal reactions
- Pseudoseptic arthritis
- Right shoulder joint pain
- Rotator cuff syndrome
- Shoulder joint effusion
- Synovitis/ tenosynovitis
- Tendinitis/ tendinosis/ tendonitis/ tendinopathy
- Torn rotator cuff
- Other

76R-1). List the diagnosis codes from Q75R.

76R-2).

76R-3).

76R-4).

76R-5).

76R-6).

76R-7).

76R-8).

76R-9).

76R-10).

77R. Did the shoulder injury occur in the RIGHT arm Yes

in which a vaccine was injected? No

Unknown

77R-1). Please copy and paste the relevant statement from the chart notes on the RIGHT shoulder injury.

78R. Did the RIGHT symptoms of shoulder injury begin Yes within the first 7 days following vaccination? No

No, but increased severity of pre-existing (Please be sure to use the most complete and reliable symptoms in the first 30 days

information to answer this question when there are Possible

discrepancies with more than one source.) Unknown

78R-1). Please copy and paste the relevant statement from the chart notes on the shoulder injury symptom onset.

79R. Did the RIGHT shoulder symptoms persist more Yes

than 30 days from the date of vaccination? No

Possible

(Please be sure to use the most complete and accurate Unknown information to answer this question when there are

discrepancies with more than one source.)

79R-1). Please copy and paste the relevant statement from the chart notes on the RIGHT shoulder injury symptom duration.

80R. What was the cause of the RIGHT shoulder injury? Vaccine

Incident (e.g., fall, auto accident)

Exercise (e.g., exercise, sports)

(Please select as many causes as documented.) Daily activity (e.g., overuse, lifting a heavy

item, work-related injury, side sleeping)

Other medical conditions (e.g., arthritis, chest pain radiating to the shoulder)

Unknown (e.g., no explicit cause, insidious/aggravating factors- 'worse with exercise')

80R-1). Please copy and paste the relevant statement from the chart notes on the cause of RIGHT shoulder injury (for all of the above responses).

81R. Does it look like a SIRVA case? Yes

No

Possible Unknown

75L. Did a true LEFT shoulder injury occur? (from Shoulder injury diagnosis code AND

the following) physician/patient mention of shoulder symptoms

Shoulder injury diagnosis code with no mention of

- Adhesive capsulitis/ frozen shoulder shoulder symptoms by physician/patient
- Bone erosion Shoulder symptoms mentioned by physician/patient
- Bursitis but no shoulder injury diagnosis code
- Humerus fractures No shoulder injury diagnosis code or
- Impingement physician/patient mention of shoulder symptoms
- Left shoulder joint pain
- Osteitis
- Osteolysis
- Osteonecrosis
- Periosteal reactions
- Pseudoseptic arthritis
- Right shoulder joint pain
- Rotator cuff syndrome
- Shoulder joint effusion
- Synovitis/ tenosynovitis
- Tendinitis/ tendinosis/ tendonitis/ tendinopathy
- Torn rotator cuff
- Other

76L-1). List the diagnosis codes from Q75L.

76L-2).

76L-3).

76L-4).

76L-5).

76L-6).

76L-7).

76L-8).

76L-9).

76L-10).

77L. Did the shoulder injury occur in the LEFT arm in Yes

which a vaccine was injected? No

Unknown

77L-1). Please copy and paste the relevant statement from the chart notes on the LEFT shoulder injury.

78L. Did the symptoms of the LEFT shoulder injury Yes

begin within the first 7 days following vaccination? No

No, but increased severity of symptoms (Please be sure to use the most complete and reliable Possible

information to answer this question when there are Unknown discrepancies with more than one source.)

78L-1). Please copy and paste the relevant statement from the chart notes on the LEFT shoulder injury symptom onset.

79L. Did the symptoms of LEFT shoulder injury persist Yes

more than 30 days from the date of vaccination? No

Possible

(Please be sure to use the most complete and accurate Unknown information to answer this question when there are

discrepancies with more than one source.)

79L-1). Please copy and paste the relevant statement from the chart notes on the LEFT shoulder injury symptom duration.

80L. What was the cause of the LEFT shoulder injury? Vaccine

Incident (e.g., fall, auto accident)

(Please select as many causes as documented.) Exercise (e.g., exercise, sports)

Daily activity (e.g., overuse, lifting a heavy item, work-related injury, side sleeping) Other medical conditions (e.g., arthritis, chest pain radiating to the shoulder)

Unknown (e.g., no explicit cause, insidious/aggravating factors- 'worse with exercise')

80L-1). Please copy and paste the relevant statement from the chart notes on the cause of the LEFT

shoulder injury (for all of the above responses).

81L. Does it look like a SIRVA case?

Yes

No

Possible Unknown

**I. GENERAL COMMENTS**

82. Do you have any questions or comments not addressed in the form? Please enter them here.
