# Supplementary material for: Identifying Cases of Shoulder Injury Related to Vaccine Administration (SIRVA) in the United States: Development and Validation of a Natural Language Processing Method
Source: JMIR Public Health Surveill. 2022 May 24;8(5):e30426. doi: 10.2196/30426 (PMC9175103; doi:10.2196/30426)
Supplement: Multimedia Appendix 3 [file publichealth_v8i5e30426_app3.docx]

**Appendix 3. Sample extracted temporal expressions**

**Identifying Cases of Shoulder Injury Related to Vaccine Administration (SIRVA) in the United States: Development and Validation of a Natural Language Processing Method**

Chengyi Zheng^1^, PhD, Jonathan Duffy^2^, MD, In-Lu Amy Liu^1^, MS, Lina S. Sy^1^, MPH, Ronald A. Navarro^3^, MD, Sunhea S. Kim^1^, MPH, Denison S. Ryan^1^, MPH, Wansu Chen^1^, PhD, Lei Qian^1^, PhD, Cheryl Mercado^1^, MPH, Steven J. Jacobsen^1^, MD, PhD

^1^ Department of Research & Evaluation, Kaiser Permanente Southern California, Pasadena, California, USA

^2^ Immunization Safety Office, Centers for Disease Control and Prevention, Atlanta, GA, USA

^3^ Kaiser Permanente South Bay Medical Center, Harbor City, California

**Corresponding Author:**

Chengyi Zheng, PhD

Department of Research and Evaluation, Kaiser Permanente Southern California

100 S Los Robles Ave, 2nd Floor,

Pasadena, CA 91101

United States

Phone: 1 626 986 8665

Email: Chengyi.X.Zheng@kp.org

Appendix 3. Sample extracted temporal expressions

- Part of the day: this morning, last night, etc.
  - left shoulder pain after a mechanical ground level fall last evening
- Date: 2/14/17, 10/1, Dec 12, etc.
  - States that her shoulder pain has been an ongoing issue since July 2016
- Weekday: Wednesday, last Monday, yesterday, etc.
  - Was changing his tire on Friday and heard a popping noise in his left shoulder, now has pain with decreased ROM.
- Month: September, etc.
  - left shoulder pain Onset w/ reaching back for seatbelt in Oct
- Season: Spring, Summer, Christmas, etc.
  - I injured my (left) shoulder in a fall last summer (2016)
- Duration: for 3 weeks, x several days, etc.
  - shoulder pn x 2 mo
  - pain, loss of motion left shoulder the past 10 years
  - Who had a fall 2-3 weeks ago - hit the right shoulder against a wall and pain getting worse

Some other temporal data were not extracted because they could not be used to infer the onset timing or duration of the shoulder injury:

- indicates symptom history but without an exact onset date. For example, "patient had been bothered with left shoulder pain"; "shoulder pain (chronic)".
- describes frequency (e.g. "every 4 weeks") or future event (e.g. "next 2 days").
