# Supplementary material for: Identifying Cases of Shoulder Injury Related to Vaccine Administration (SIRVA) in the United States: Development and Validation of a Natural Language Processing Method
Source: JMIR Public Health Surveill. 2022 May 24;8(5):e30426. doi: 10.2196/30426 (PMC9175103; doi:10.2196/30426)
Supplement: Multimedia Appendix 4 [file publichealth_v8i5e30426_app4.docx]

**Appendix 4. Trigger phrases for identifying causal relationships**

**Identifying Cases of Shoulder Injury Related to Vaccine Administration (SIRVA) in the United States: Development and Validation of a Natural Language Processing Method**

Chengyi Zheng^1^, PhD, Jonathan Duffy^2^, MD, In-Lu Amy Liu^1^, MS, Lina S. Sy^1^, MPH, Ronald A. Navarro^3^, MD, Sunhea S. Kim^1^, MPH, Denison S. Ryan^1^, MPH, Wansu Chen^1^, PhD, Lei Qian^1^, PhD, Cheryl Mercado^1^, MPH, Steven J. Jacobsen^1^, MD, PhD

^1^ Department of Research & Evaluation, Kaiser Permanente Southern California, Pasadena, California, USA

^2^ Immunization Safety Office, Centers for Disease Control and Prevention, Atlanta, GA, USA

^3^ Kaiser Permanente South Bay Medical Center, Harbor City, California

**Corresponding Author:**

Chengyi Zheng, PhD

Department of Research and Evaluation, Kaiser Permanente Southern California

100 S Los Robles Ave, 2nd Floor,

Pasadena, CA 91101

United States

Phone: 1 626 986 8665

Email: Chengyi.X.Zheng@kp.org

**Trigger phrase before the causal phrase**

*[at above in near] site of*

*[blame* attribute* complain*]*

*[compatible consistent] [for with]*

*[got given getting]*

*[reaction rxn react]*

*[was been]*

*after*

*[associated association] with*

*[because cause due]*

*because of*

*c / w*

*cause of injury*

*caused by*

*complication* of*

*consequence* of*

*doing*

*due to*

*during*

*etiology*

*evidence [for of]*

*except for*

*experienced*

*following*

*from*

*had*

*manifestation of*

*mark* of*

*owing to*

*post*

*receive**

*relate* to*

*result* [from of]*

*s / p*

*[second secondary] to*

*since*

*started with*

*status post*

*status-post*

*was from*

*was given*

[ ] indicates selection of one of the words within the bracket

* indicates capture of all the morphological variants of that term

Example: “Pt presents with a chief complaint of left shoulder pain, no weakness that began 6 months ago after flu shot.” In the above example, “after” is the trigger phrase, and “flu shot” is the causal phrase.

**Trigger phrase after the causal phrase**

*causing*

*resulting*

*next [day week]*

*[after later] /word [day week]*

*[day* week*] [after later]*

*was [given administered injected]*

*[reaction rxn react]*

*related*

/word means any word.

Example:

“Received the flu shot on December 13 and the next day she reported she was unable to move her arm.”

In the above example, “next day” is the trigger phrase, and “flu shot” is the causal phrase.
