# Supplementary material for: Identifying Cases of Shoulder Injury Related to Vaccine Administration (SIRVA) in the United States: Development and Validation of a Natural Language Processing Method
Source: JMIR Public Health Surveill. 2022 May 24;8(5):e30426. doi: 10.2196/30426 (PMC9175103; doi:10.2196/30426)
Supplement: Multimedia Appendix 5 [file publichealth_v8i5e30426_app5.docx]

**Appendix 5. Terminology for causes of shoulder injury other than vaccination**

**Identifying Cases of Shoulder Injury Related to Vaccine Administration (SIRVA) in the United States: Development and Validation of a Natural Language Processing Method**

Chengyi Zheng^1^, PhD, Jonathan Duffy^2^, MD, In-Lu Amy Liu^1^, MS, Lina S. Sy^1^, MPH, Ronald A. Navarro^3^, MD, Sunhea S. Kim^1^, MPH, Denison S. Ryan^1^, MPH, Wansu Chen^1^, PhD, Lei Qian^1^, PhD, Cheryl Mercado^1^, MPH, Steven J. Jacobsen^1^, MD, PhD

^1^ Department of Research & Evaluation, Kaiser Permanente Southern California, Pasadena, California, USA

^2^ Immunization Safety Office, Centers for Disease Control and Prevention, Atlanta, GA, USA

^3^ Kaiser Permanente South Bay Medical Center, Harbor City, California

**Corresponding Author:**

Chengyi Zheng, PhD

Department of Research and Evaluation, Kaiser Permanente Southern California

100 S Los Robles Ave, 2nd Floor,

Pasadena, CA 91101

United States

Phone: 1 626 986 8665

Email: Chengyi.X.Zheng@kp.org

**Note:**

Each line represents one entry of the terms

[] indicates selection of one of the words within the bracket

* indicates capture of all the morphological variants of that term

The top-level causes are:

- cause_accident
- cause_work
- cause_other_medical_conditions
- cause_exercise
- cause_daily_activity
- cause_unknown

**cause_accident**

abrasion

accident*

accidental fall

airbag

auto accident

bite

blow

car

car accident

CHP

collided

collision

dislocated

dislocation

driving

fall*

FALL:

hit*

incident

landing

landed

mechanical fall

motor vehicle accident

motorcross

motorvehicle accident

motor-vehicle accident

MVA

passenger

punctured

rear ended

rear-end

rearended

rear-ended

separation

slip*

struck

t bone

t boned

t-bone

t-boned

trauma*

traumatic

tripped

twisted

vehicle accident

**cause_work**

job

work

workers compensation

**cause_other_medical_conditions**

abrasion

arithritis

arthritic

arthritis

athritis

back pain

birth

cervical spondylosis

CVA

degenerative

djd

fibromyalgia

mastectomy*

MCA

multifactorial

neck pain

neckpain

neuritis

neuropathy

OA

osteoarthritic

osteoarthritis

polyarthritis

radiating pain

radicular

radiculitis

radiculopathy

referred pain

sciatica

spondyloarthropathy

stroke

surgery

whiplash

whiplashed

**cause_exercise**

aerobics

archer

archery

arena

arrow

athlete

athletics

axel

badminton

ball

baseball

basketball

bat

baton

batter

batting

bench press

biathlon

bicycle

bicycling

bike

biking

billiards

bobsleigh

bocce

boomerang

boules

bow

bowler

bowling

boxer

boxing

bronze medal

bunt

canoe

canoeing

catch

catcher

champion

championship

cleats

club

coach

compete

competing

competition

competitor

crew

cricket

croquet

cross country

curling

cycle

cycling

cyclist

dart

dartboard

deadlifting

decathlon

defense

diamond

discus

dive

diver

diving

dodgeball

doubleheader

dugout

épée

equestrian

fencing

field hockey

fielder

fielding

figure skating

fishing

fitness

football

free throw

frisbee

game*

geocaching

goalie

gold medal

golf

golfer

golfing

got hit

gym

gymnasium

gymnast

gymnastics

hammer throw

hand stand

handball

hang gliding

hardball

helmet

heptathlon

high jump

hitter

hockey

hole-in-one

hoop

horseshoes

huddle

hurdle

ice hockey

ice rink

ice skates

ice skating

infield

infielder

inline skates

inning

jai-alai

javelin

jog

jogger

judo

jump

jump rope

jumper

jumping

karate

kayak

kayaker

kayaking

kickball

kite

kung fu

lacrosse

lawn bowling

league

lift* weight*

long jump

luge

lutz

mallet

martial art*

mitt

offense

ollie

Olympics

orienteering

outfield

outfielder

overhead press

paddle

paddleball

paddling

paintball

parallel bar*

parasailing

parkour

pentathlon

pickleball

ping pong

pitch

pitcher

play*

playground

player

playoffs

pogo stick

pole

pole vault

polo

pool

puck

pull up*

push* up*

quarterback

quiver

race

racer

racewalking

racing

racket

racquetball

rafting

riding

rink

rock climbing

roller-blading

roller blading

rollerblade*

rollerskate*

roller skates

roller skating

roller-skates

roller-skating

row

rower

rowing

rqcquet

rugby

run

runner

running

sailing

scoot*

scuba

scull

sculling

shortstop

shot put

silver medal

skate

skating rink

skeleton

ski

skier

skiing

slalom

sled*

sledder

snorkeling

snowboard

snowboarder

snowboarding

snowshoeing

soccer

softball

somersault

speed skating

sport*

sportsmanship

squash

stadium

strike

sumo wrestling

surf*

surfer

swim*

swimmer

table tennis

taekwondo

taiko

tennis

tetherball

throw

throwing

toboggan

track and field

trampoline

triathlete

triathlon

tricycle

triple jump

triple play

tug of war

ultramarathon

ultramarathoner

umpire

unicycle

unicyclist

vault

vaulter

vaulting

volley

volley ball

volleyball

wakeboarding

water polo

water ski

water skier

water skiing

weightlifter

weightlifting

weight lifting

weight training

weights

wicket

windsurfer

windsurfing

work* out

workout

wrestler

wrestling

yoga

**cause_daily_activity**

carry*

cleaning

grab*

heavy

lift*

massage

overuse*

paint*

pick*

poor posture

reaching

repetitive [injury* injury- injruy injurys injuries injurious injured injure injuring]

repetitive [motion* use* strain* lift* type*]

wake* up

**cause_unknown**

[no none]

[unsure unknown unclear uncertain undertermined unknwon]

[insidious insidiuos incidious]

gradual

[N/A na]
